# Engineered metallic hybrid nanozyme for advanced inflammatory disease therapy

**DOI:** 10.1016/j.mtbio.2025.102572

**Published:** 2025-11-19

**Authors:** Xueqian Xia, Xiang Liu, Yue Gao, Jiatong Lin, Shuangxue Pan, Weijian Cheng, Sheng Huang, Xingyue Liu, Jia-Wei Shen, Wei Duan

**Affiliations:** a School of Pharmacy, Hangzhou Normal University, Hangzhou, 311121, Zhejiang, China; b Ecology and Health Institute, Hangzhou Vocational & Technical College, Hangzhou, 310032, Zhejiang, China; c Zhejiang Provincial Key Laboratory of Anti-Cancer Chinese Medicines and Natural Medicines, Engineering Laboratory of Development and Application of Traditional Chinese Medicines, Collaborative Innovation Center of Traditional Chinese Medicines of Zhejiang Province, Hangzhou Normal University, Hangzhou, 311121, Zhejiang, China; d State Key Laboratory of Silicon and Advanced Semiconductor Materials, Zhejiang University, Hangzhou, 310027, China; e State Key Laboratory of Molecular Engineering of Polymers (Fudan University), Shanghai, 200438, China

**Keywords:** Metallic hybrid nanozyme, Inflammatory diseases, ROS scavenging, Combined therapy, Rational design

## Abstract

Inflammation poses significant therapeutic challenges due to the limitations of conventional treatments. Nanozymes, merging nanomaterial properties with enzymatic catalysis, offer promising alternatives for inflammation therapy. Among them, metallic nanozymes have attracted considerable attention due to their tunable physicochemical properties and multi-therapeutic potential. However, single-metal nanozymes suffer from insufficient catalytic efficiency, high therapeutic dosage requirements, and limited functionality, which severely hinder their clinical translation. Recent advances in metallic hybrid nanozymes, which integrate the advantages of different metallic elements, have enabled synergistic enhancement of catalytic performance and cascade effects, offering more effective solutions for inflammation treatment. In this review, we first systematically outline the fundamental principles, molecular mechanisms, and current therapeutic approaches for inflammation. Subsequently, we comprehensively discuss the definition, catalytic mechanisms, and rational design strategies of nanozymes, with a focus on the common synthesis methods, activity regulation mechanisms, computer-aided design strategies, and combination therapy approaches for metallic hybrid nanozymes. Furthermore, we provide an in-depth evaluation of the applications of metallic hybrid nanozymes in treating various inflammatory diseases. Finally, we critically analyze the key scientific challenges and future directions in the clinical translation of metallic hybrid nanozymes. This work provides a comprehensive framework for developing next-generation nanozyme therapeutics, bridging fundamental research and clinical applications in inflammation management.

## Highlights

• Inflammation poses significant therapeutic challenges due to the limitations of conventional treatments.
• Metallic hybrid nanozymes surpass single-metal systems by synergistically integrating different metals.
• These nanozymes show superior anti-inflammatory effects across various diseases by multiple combined mechanisms.
• A comprehensive bridge for metallic hybrid nanozyme and its clinical translation in inflammation management is provided.

## Graphical abstract

**Figure:**
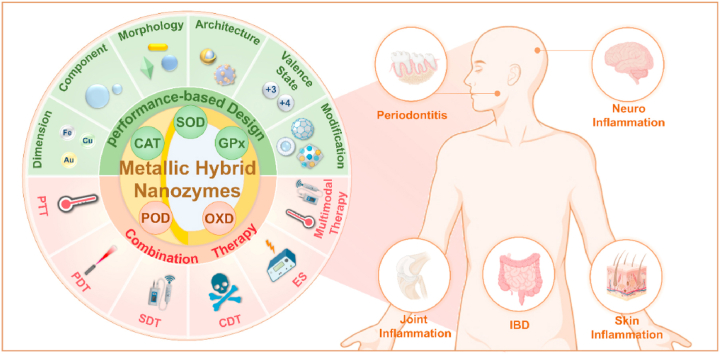

## 1 Introduction

Inflammation is an essential defense mechanism for restoring homeostasis [1]. However, when excessive or chronic, it contributes to numerous inflammatory and autoimmune diseases [2], posing a significant global health burden [3]. In the U.S. alone, chronic conditions—largely fueled by persistent inflammation—are projected to affect 129 million adults by 2024, accounting for 90 % of the nation's $4.1 trillion in annual healthcare expenditures [4]. This immense burden underscores that conventional therapies are often inadequate, necessitating the development of more effective novel therapies [5].

Natural enzymes are an important class of biological catalysts found in living organisms, typically composed of proteins or RNA, with intricate structures and high substrate specificity [6,7]. However, high costs, poor bioavailability, potential antigenicity, and low stability under pathological conditions limit the clinical application of natural enzymes [8].With the rise of nanotechnology, nanozymes have emerged as a new biomimetic catalytic material that not only mimics the catalytic functions of natural enzymes but also possesses the physical and chemical properties of nanomaterials. They can leverage their own nanoscale characteristics, such as excellent tunability and ease of modification [9,10], making them highly promising for applications in the inflammation therapy field [11,12]. Metal nanozymes are biomimetic catalytic materials inspired by the metal active centers in natural enzymes. The central metal ions not only effectively promote substrate adsorption and electron transfer but also serve as the core sites for redox reactions, thereby conferring enzyme-like activity [13,14]. Furthermore, the distinctive physical characteristics of metal nanoparticles, including their capacity for light absorption, electricity, sound, and magnetism, render them well-suited for integration with a range of treatment modalities, such as photodynamic therapy (PDT) and sonodynamic therapy (SDT) [15,16].

However, conventional single-metal nanozymes often encounter issues such as inadequate catalytic activity, unsatisfactory selectivity, and restricted control methodologies in intricate biological environments [17,18]. In recent years, metallic hybrid nanozymes have become a subject of considerable research interest owing to their distinctive structural characteristics and synergistic mechanisms. Compared to their single-metal counterparts, metallic hybrid nanozymes enable precise modulation of electronic structures and surface properties through the integration of diverse metal elements, resulting in breakthrough improvements in both catalytic activity and specificity. This unique hybrid design not only optimizes the electronic structure of active centers through the synergistic effects of bimetallic or multimetallic compositions, significantly enhancing single enzyme-mimicking activity [19], but also allows the integration of multiple enzymatic activities to execute efficient cascade catalytic reactions within inflammatory microenvironments, enabling thorough reactive oxygen species (ROS) scavenging. Furthermore, the incorporation of a secondary metal can impart novel physical properties to the system, such as enhanced photothermal conversion efficiency, thereby creating powerful synergistic effects between catalytic therapy and physical modalities like photothermal or sonodynamic therapy, ultimately improving overall therapeutic outcomes [20]. More importantly, the exceptional catalytic efficiency of metallic hybrid nanozymes allows them to achieve desired therapeutic effects at lower dosages. Combined with their designable targeting capability, this significantly minimizes off-target effects and reduces the risk of adverse reactions, demonstrating substantial clinical translation potential [21]. These unique advantages position metallic hybrid nanozymes as highly promising candidates for addressing multifaceted clinical challenges, particularly in the realms of targeted anti-inflammatory and antioxidant therapies.

The rational design of metallic hybrid nanozymes is crucial for their catalytic activity. The development of nanozymes with multiple functions is possible through the achievement of coordinated coordination and electron transfer between different metal centers. The greater complexity of both composition and mechanism in metallic hybrid nanozymes means that their rational design and efficient synthesis are more complex and challenging. Nevertheless, their superior performance, attributable to rational design, renders them highly promising candidates for the treatment of inflammation. It is important to acknowledge the significant role of computational science in advancing theoretical investigations. Such computational approaches can help elucidate potential simulated mechanisms of metallic nanozymes, thereby establishing a more advanced and efficient foundation for the rational design of these metallic enzymes [20]. In addition to enhancing catalytic performance, metallic hybrid nanozymes have the capacity to fulfil a number of functions in the treatment of inflammation. For instance, a rationally designed Pt@PCN222-Mn metallic hybrid system has been shown to possess the capability of eliminating excess free radicals through cascade reactions, thereby attenuating inflammatory responses, regulating immune cell activity, and enhancing the local microenvironment [22]. These characteristics confer significant application potential for metallic hybrid nanocatalysts in the treatment of various chronic inflammatory diseases, including arthritis, neuroinflammatory conditions, and skin inflammation. As demonstrated in recent reviews, there is a growing body of literature exploring the practical applications of metallic hybrid nanozyme in detection and medical fields [[23], [24], [25]]. However, there is a paucity of systematic research on the regulatory mechanisms of metallic hybrid nanozyme and their applications in various inflammatory diseases.

**Fig. 1:**
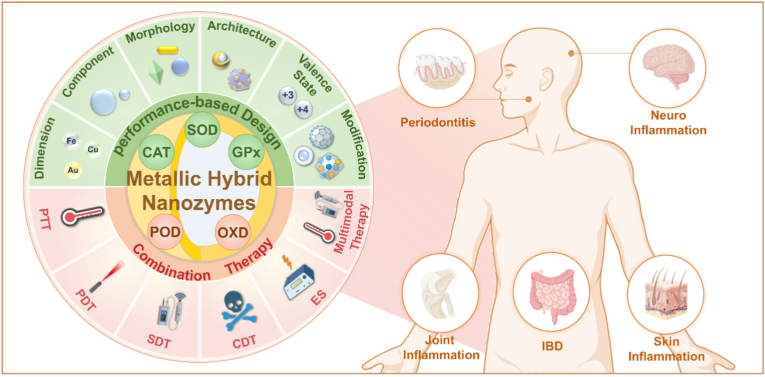
Metallic hybrid nanozymes for treatment of various inflammatory diseases through rational design and combination therapy. (Created with BioRender.com).

The present review focuses on the characteristics of metallic hybrid nanozymes, with a particular emphasis on the potential mechanisms by which metallic hybrid nanozymes regulate inflammation through multiple pathways (Fig. 1). Notably, the review argues for the application prospects and design concepts of nanozymes in the biomedical field by discussing the corresponding treatment strategies and methods for metallic hybrid nanozymes in typical inflammatory diseases in recent years. It is hoped that this review will provide researchers with a more profound understanding of the subject, and act as a reference point and a source of inspiration for advancing the clinical application of metallic hybrid nanozymes.

## 2 Inflammation

### 2.1 Introduction of inflammation

Inflammation is a core defense mechanism for maintaining the homeostasis of the body's internal environment. It is essentially an adaptive response of the immune system to pathogen invasion, tissue damage, or endogenous danger signals, aimed at eliminating harmful factors and restoring the body's balance [1]. Inflammation promotes the production of inflammatory factors (cytokines) by immune cells to combat foreign substances, clear harmful agents from the organism, and stimulate tissue repair [26]. This process is typically accompanied by the signs of redness, swelling, heat, and pain, as well as the formation of exudate and the occurrence of systemic reactions.

Inflammation has a dual nature. A moderate inflammatory response is crucial for maintaining homeostasis under harmful stimuli and helps the body repair after infection or injury. However, an excessive or persistent inflammatory response disrupts homeostasis, leading to tissue dysfunction and even organic damage to organs. Based on its duration, inflammation can be classified into acute and chronic inflammation. Acute inflammation is a short-term response of the body to specific stimuli (such as skin trauma, bacterial infection, ischemia-reperfusion injury, etc.), usually characterized by neutrophil infiltration and rapid resolution, being self-limiting and relatively short in duration [27]. Most acute inflammations can resolve within 7–10 days. If the stimulating factor persists or immune regulation is abnormal, it may develop into chronic inflammation (such as periodontitis, arthritis, etc.). Chronic inflammation is a long-term, persistent defensive response characterized by lymphocyte and macrophage infiltration, tissue destruction, repair (often with fibrosis), and neovascularization, which can last for months or even years. Uncontrolled chronic inflammatory responses can lead to progressive tissue damage, DNA damage, and abnormal cell proliferation due to continuous immune activation, posing a serious threat to human health [28].

Notably, inflammation itself creates a distinct pathological microenvironment. Compared to normal tissues, inflamed sites typically exhibit an acidic pH (∼6.4), overexpression of inflammation-related enzymes such as cyclooxygenase, and heightened oxidative stress characterized by elevated levels of ROS and reactive nitrogen specie. This microenvironment activates nuclear factor kappa-B (NF-κB) and other signaling pathways, promoting the release of pro-inflammatory cytokines and exacerbating tissue damage.

### 2.2 Mechanism of inflammation

The inflammatory response is a core defense mechanism of the body against external stimuli or internal damage. Its occurrence and development are highly complex, involving dynamic changes in multiple regulatory factors, cell types, signaling pathways, as well as metabolic and oxidative stress states [29]. Its onset and regulation depend on the synergistic action of positive and negative regulatory mechanisms to ensure that inflammation is moderately activated and timely terminated.

The initiation and amplification of inflammation begin with the activation of the innate immune system. During this cascade, pattern-recognition receptor—exemplified by the abundantly expressed macrophage Toll-like receptors that sense PAMPs and DAMPs—ignite pivotal downstream signaling axes such as the NLRP3 inflammasome, NF-κB, the mitogen-activated protein kinase (MAPK) and JAK-STAT pathways. The convergence of these signals promotes inflammasome assembly and unleashes a surge of pro-inflammatory cytokines, including TNF-α, IL-6 and IL-1 [30,31]. Moreover, the inflammatory response is orchestrated by the interplay of multiple cell types, including dendritic cells, regulatory T cells, macrophages and neutrophils. After capturing antigens at the inflamed site, dendritic cells present them to T cells, thereby initiating an adaptive immune response. During the T-cell-mediated phase, T cells activate macrophages through direct contact or by secreting cytokines such as IFN-γ, driving them toward the classically activated M1 pro-inflammatory phenotype. These M1 macrophages phagocytose pathogens and release large quantities of inflammatory mediators—processes that are prototypical in rheumatoid arthritis and acute kidney injury [32,33]. Neutrophils constitute the principal effector cells during acute inflammation. They contribute by phagocytosing pathogens and generating ROS, and they also release vascular endothelial growth factor (VEGF), thereby promoting angiogenesis and tissue remodeling [34,35]. Locally produced pro-inflammatory cytokines such as IL-1, TNF and IL-6 modulate the tissue microenvironment, inducing vasodilation, increased vascular permeability, neutrophil extravasation and plasma leakage into the infected locus. Once these mediators reach a critical systemic concentration, the inflammatory reaction may spread beyond the local site, giving rise to systemic manifestations including fever and acute-phase responses [36].

To prevent excessive inflammation and attendant tissue damage, the immune system deploys multiple negative-feedback mechanisms. Anti-inflammatory cytokines such as IL-10 and TGF-β, together with regulatory T cell, suppress the production of pro-inflammatory mediators. Moreover, exposure to Th2-derived cytokines IL-4 and IL-13 drives macrophages to switch from the pro-inflammatory M1 phenotype to the alternatively activated, anti-inflammatory M2 phenotype [33]. Collectively, these mechanisms dampen inflammatory signaling, reverse tissue injury, and promote the resolution of inflammation and tissue repair.

### 2.3 The role of ROS in modulating inflammation and relevant pathways

ROS, free-radical derivatives of oxygen, mainly include superoxide anion (•O_2_^-^), hydrogen peroxide (H_2_O_2_), hydroxyl radical (•OH), and singlet oxygen (^1^O_2_). Due to the presence of unpaired electrons, ROS exhibit high chemical reactivity [37]. Most ROS are generated from the mitochondrial electron transport chain. During cellular oxidation, most electrons are safely transferred from donors to acceptor molecules through various redox pathways. However, when electrons leak prematurely from complexes, they mediate the single-electron reduction of oxygen to O_2_•^-^, which is then converted into H_2_O_2_. When mitochondrial membrane permeability increases, ROS generated by METC in mitochondria can be released into the cytosol, inducing inflammation through signal transduction or toxic damage to macromolecules. Additionally, ROS are also produced as by-products of various enzymes, such as NADPH oxidases and lipoxygenase.

ROS act as secondary messengers in various physiological processes, including signal transduction, gene expression, angiogenesis, and cell migration, playing essential roles in maintaining cellular redox homeostasis and tissue repair. At physiological levels, ROS function as signaling molecules that regulate redox balance, promote angiogenesis, and facilitate wound healing. However, when ROS accumulate excessively, they induce oxidative stress, leading to biochemical changes such as protein carbonylation, lipid peroxidation, and damage to lipid membranes, proteins, and DNA, ultimately resulting in cellular senescence or apoptosis, and contributing to a range of inflammatory diseases [38].

In addition to direct cytotoxicity, ROS can also modulate inflammation under pathological conditions through reversible oxidative modifications of signaling molecules, acting as key regulators in the initiation and progression of inflammation, and affecting multiple inflammatory signaling pathways, thereby exacerbating the inflammatory response [39]. Noteworthy, ROS influence mitochondrial DNA (mtDNA) release, which plays a crucial role in inflammation [40]. During mitochondrial oxidative phosphorylation, excessive ROS accumulation can result in mitochondrial membrane depolarization and the opening of mitochondrial permeability transition pores (mPTP), facilitating the release of mtDNA [41] Released mtDNA acts as damage-associated molecular patterns (DAMPs), which activate inflammatory pathways by interacting with DNA sensors such as cGAS, TLR9, and NLRP3, leading to the secretion of pro-inflammatory cytokines like IL-1β and sustaining chronic inflammation. Mitochondrial dysfunction can also cause the transport of mtDNA via exosomes, inducing systemic immune responses [42]. For example, mtDNA released during pyroptosis is packaged in exosomes and can trigger systemic inflammation in neighboring cells, amplifying the inflammatory response [43]. ROS can modulate the expression profiles of inflammation-related genes by regulating histone modifications and DNA methylation status, thereby shaping long-term inflammatory phenotypes [44]. These findings provide new perspectives for understanding the persistence mechanisms of chronic inflammation. A systemic understanding of how ROS regulate these signaling pathways is essential for revealing the complex mechanisms of inflammation and developing novel anti-inflammatory strategies.

#### 2.3.1 NLRP3 inflammasome pathway

NLRP3 inflammasome is one of the most representative inflammasomes, playing a crucial role in the initiation and regulation of inflammatory responses. It consists of the NLRP3 protein, the adaptor protein ASC, and the effector protein caspase-1. Upon activation, the inflammasome recruits and activates caspase-1, promoting the maturation and secretion of IL-1β and IL-18, further amplifying the inflammatory response [45]. Dysregulation of NLRP3 is closely associated with the pathogenesis of various inflammatory diseases, such as osteoarthritis (OA) and Alzheimer's disease (AD) [46,47]. Zhou et al. demonstrated that under oxidative stress conditions, ROS oxidize thioredoxin, which leads to the release of thioredoxin-interacting protein (TXNIP). TXNIP then binds to NLRP3, triggering inflammasome activation [48]. Additionally, studies have shown that ROS, particularly mitochondrial-derived ROS (mtROS), are key upstream mediators in the activation of the NLRP3 inflammasome. An et al. found that the pathogen pattern recognition receptor Omp34 from Acinetobacter baumannii can induce NLRP3 inflammasome activation through mtROS [49]. Furthermore, Shimada et al. discovered that mtROS can oxidize mitochondrial DNA (mtDNA), and the oxidized mtDNA, released into the cytosol, directly interacts with NLRP3, serving as an endogenous danger signal to activate the inflammasome [50].

#### 2.3.2 NF-κB pathway

The NF-κB signaling pathway is a central mediator in the transcription of pro-inflammatory genes and the regulation of immune cell functions [51]. It is critical for the transcription of several pro-inflammatory genes, such as TNF-α, IL-1β, IL-6, IL-12, and COX-2 [52]. In its resting state, NF-κB is bound to the inhibitor protein IκB, which retains it in the cytoplasm. Upon activation by TLR or TNF receptors, IκB is phosphorylated and degraded, allowing the released NF-κB to enter the nucleus and initiate the expression of various inflammatory genes, including TNF-α and IL-6. NF-κB not only participates in the transcription of pro-inflammatory genes but also plays a crucial role in regulating immune cell functions. It promotes the transcription of M1 macrophages and induces the expression of pro-inflammatory cytokines such as TNF-α and IL-1β, thereby exacerbating the inflammatory response [53]. Additionally, NF-κB can promote the formation of the NLRP3 inflammasome, further amplifying the inflammatory signal. ROS exacerbate this inflammatory response by inducing NF-κB activation, a process particularly evident in various inflammatory diseases, such as IBD and rheumatoid arthritis [54]. In 1991, Schreck et al. first demonstrated that direct addition of H_2_O_2_ to the culture medium could activate NF-κB [55]. Subsequently, Lee et al. found that the antioxidant L-2-oxo-thiazolidine-4-carboxylic acid significantly inhibited NF-κB nuclear translocation and the transcription of downstream pro-inflammatory genes, thereby confirming the role of ROS in mediating NF-κB activation [56].

#### 2.3.3 MAPK pathway

The MAPK family comprises extracellular signal-regulated kinase (ERK), stress-activated protein kinase (SAPK), and p38, with all three branches collaboratively regulating the inflammatory response [57,58]. The substrate pocket of p38 MAPK, upon binding with proteins or peptides and ATP, activates MAPK, leading to its phosphorylation and the subsequent activation of kinases or nuclear proteins, including transcription factors. In response to pro-inflammatory cytokines (TNF-α and IL-1β) and cellular stressors (such as hypoxic or oxidative stress), the SAPK and p38 signaling cascades are triggered, playing a critical role in the pathogenesis of inflammatory diseases like rheumatoid arthritis [52].

Studies have shown that ROS can amplify inflammatory signals by either oxidatively modifying upstream proteins in the MAPK signaling pathway or inhibiting the activity of MAPK phosphatases (MKPs), leading to sustained phosphorylation of MAPK. This process results in the overproduction of pro-inflammatory mediators such as IL-6 and TNF. For instance, Hsieh et al. found that ROS activate the p38 MAPK pathway by forming a complex with ASK1 through the oxidation of thioredoxin (Trx), thus promoting inflammation in hepatocytes [59]. Additionally, Bulua et al. discovered that ROS scavengers effectively reduced the persistent phosphorylation levels of JNK and p38 in a TNFR1-associated periodic syndrome (TRAPS) model [60].

#### 2.3.4 JAK/STAT pathway

The JAK-STAT pathway is responsible for transmitting extracellular signals into the cell nucleus and is composed of three main components: tyrosine kinase-associated receptors, JAKs, and STATs [61]. Upon binding of cytokines, such as IL-6, to their receptors, JAK kinases are activated, leading to phosphorylation of STAT proteins. The phosphorylated STAT proteins dimerize and enter the nucleus, where they directly regulate the expression of target genes, thereby enhancing inflammation through a positive feedback mechanism [62]. The JAK/STAT signaling pathway is involved in the pathogenesis of many inflammatory and autoimmune diseases, such as rheumatoid arthritis, psoriasis, and inflammatory bowel disease (IBD) [63].

Studies have shown that ROS can enhance the inflammatory response by directly activating JAK kinases or by oxidizing and phosphorylating STAT proteins to increase their transcriptional activity. For example, Choi et al. found that ROS enhanced the pro-inflammatory response induced by lipopolysaccharide by activating STAT3 [64]. Furthermore, by promoting the phosphorylation of STAT3, ROS also enhance the activation of M1 macrophages, further exacerbating the inflammatory response.

#### 2.3.5 Other pathways

Nuclear factor erythroid 2-related factor 2 (Nrf2) is a pivotal transcription factor involved in the cellular response to oxidative stress. It activates a series of antioxidant genes that aid in the clearance of ROS and other harmful molecules. In inflammation, ROS activate Nrf2 to induce the expression of antioxidant genes, alleviating oxidative damage and inflammatory responses. Studies have demonstrated that Nrf2 activation enhances the expression of antioxidant enzymes such as HO-1 and NQO1, thereby inhibiting the release of inflammatory mediators and reducing chronic inflammation. In conditions such as diabetes and neurodegenerative diseases, Nrf2 activation helps slow disease progression.

The PI3K/AKT signaling pathway plays a critical role in various biological processes, including cell growth, survival, proliferation, and metabolism. During the inflammatory response, the PI3K/AKT pathway regulates the release of cytokines and chemokines, promoting immune cell activation and enhancing inflammation. ROS can activate PI3K or AKT, thereby increasing the secretion of inflammatory mediators and modulating immune cell functions. For instance, studies have shown that ROS, through the activation of the PI3K/AKT pathway, can amplify the inflammatory response, particularly in autoimmune diseases and infections. Furthermore, this pathway also regulates cell survival and apoptosis, contributing to the development of various chronic inflammatory diseases.

**Fig. 2:**
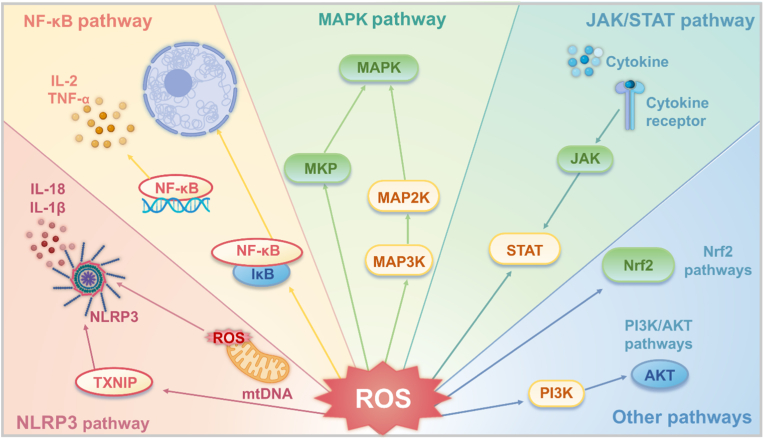
Signal transduction of ROS in inflammation. ROS play a central role in modifying various signaling molecules to regulate inflammation. They influence multiple inflammatory signaling pathways, including the NLRP3 inflammasome pathway, NF-κB pathway, MAPK pathway, JAK/STAT pathway, and other pathways. (Created with BioRender.com).

In summary, ROS serve as a central "hub" in inflammation regulation. They function as a double-edged sword, amplifying and maintaining pro-inflammatory signals via the activation of pathways such as MAPK, JAK/STAT, and PI3K/AKT, while simultaneously activating the Nrf2 pathway to trigger anti-inflammatory defense mechanisms. As such, the final outcome of inflammation (whether resolution or progression to chronic disease) is heavily influenced by the dynamic balance between these opposing signaling pathways. This makes ROS and their associated pathways critical targets for the development of new anti-inflammatory therapies (Fig. 2).

### 2.4 Treatment of inflammation

Traditional anti-inflammatory drugs have long been the cornerstone of treating inflammatory diseases. Non-steroidal anti-inflammatory drugs alleviate pain, fever, and swelling by inhibiting cyclooxygenase activity, thus blocking the synthesis of pro-inflammatory mediators like prostaglandins. Glucocorticoids and immunosuppressants exert anti-inflammatory effects by non-specifically suppressing the expression of inflammatory genes or immune cell activation pathways. Although these drugs are effective in controlling symptoms, their non-selective action mechanisms often result in side effects, including gastrointestinal damage, metabolic disorders, and systemic immune suppression, making them limited in their clinical use [65]. With the development of targeted therapies, biologics (such as TNF-α inhibitors, IL-1/IL-6 antagonists) have enhanced treatment precision by specifically blocking key inflammatory factors or immune pathways. However, challenges remain, such as complex manufacturing processes, high costs, and the need for injection administration. Furthermore, some patients may develop antibodies, leading to reduced efficacy. Notably, ROS, as central mediators in inflammatory cascades, can induce oxidative stress damage and amplify inflammatory signals.

It is worth noting that ROS, as core mediators of the inflammatory cascade, can lead to oxidative stress and amplify inflammation signals. While antioxidant therapies targeting ROS theoretically offer benefits, exogenous antioxidants (such as N-acetylcysteine) are rapidly metabolized, distributed non-specifically across tissues, and have limited ability to penetrate pathological barriers, making it challenging to achieve effective therapeutic concentrations at disease sites, limiting their clinical potential [66]. As such, there is an urgent need for novel therapeutic strategies that combine efficient ROS scavenging, lesion targeting, and microenvironment responsiveness. To address the aforementioned challenges, the development of novel nanotherapeutics with enhanced ROS-responsive and regulatory capabilities has become increasingly imperative, driven by advances in nanoscience and materials technology.

## 3 Nanozyme for inflammation regulation

Natural enzymes, despite their high substrate specificity and finely tuned structures, face significant limitations, including susceptibility to inactivation, lack of targeting ability, and difficulty in recovery, which severely restrict their use in biomedical applications like inflammation therapy [67,68]. While many types of natural enzymes exist, only a few have been successfully developed into clinical drugs. In recent years, engineered nanozymes, with their biomimetic catalytic properties and customizable responsive design, have opened new pathways to overcome these challenges. These nanomaterials mimic the catalytic activity of natural enzymes to dynamically remove excess ROS in the inflammatory microenvironment while also regulating redox signaling pathways. Additionally, they can be designed to respond to specific microenvironmental features (such as low pH and high ROS concentrations) for targeted release and can be surface-modified to cross biological barriers, including the blood-brain barrier. This multi-mechanism synergistic strategy not only addresses the non-targeted drawbacks of traditional drugs but also enhances the potential applications of engineered nanozymes in inflammation treatment due to their high stability, tunability, and scalability for large-scale production.

**Fig. 3:**
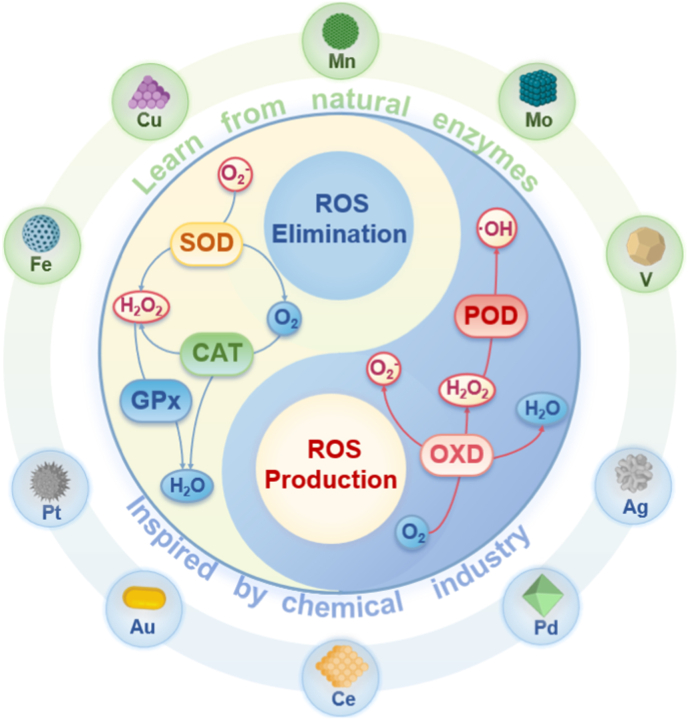
Schematic diagram of various metal nanozymes inspired by the active sites of natural enzymes and industrial chemistry, which regulate ROS through enzyme-like activity. (Created with BioRender.com).

Notably, about half of the natural enzymes are metalloproteins, where the metal atoms or metal clusters in the protein's framework serve as the central active sites for catalysis [69]. Building on this, artificial metal nanozymes have been developed to replicate these active sites and catalytic mechanisms. Transition metals, such as iron, copper, and manganese, along with their oxides, show excellent ability to simulate the natural active centers at the nanoscale. With the growing body of research, some metals traditionally used in industrial redox catalysis, including Ce, Au, Ag, and Pt, have been found to exhibit excellent enzyme-like activities, further expanding the metal nanozyme system [70]. Compared to natural enzymes, metal nanozymes not only display greater stability, but their catalytic performance can also be precisely controlled through engineering their size, morphology, and composition. They are also easier to produce at scale, modify on the surface, and reuse. Therefore, exploring the enzyme-like activities and catalytic mechanisms of metal nanozymes is a crucial foundation for advancing their application in biomedical fields (Fig. 3) [71,72].

### 3.1 Regulation by classical enzyme-like activity

Nanozymes primarily regulate inflammation by modulating ROS balance, affecting the signal transduction of key factors, and regulating metabolic processes through their enzyme-like activity. Nanozymes demonstrate dual properties: they can effectively eliminate pathogens by increasing local ROS levels, thus preventing the worsening of inflammation, while also reducing excessive intracellular ROS levels to treat inflammation-related diseases caused by ROS accumulation.

Based on their catalytic mechanisms, nanozymes can be categorized into seven types: oxidoreductases, hydrolases, transferases, isomerases, lyases, ligases, and transposases. Most nanozymes primarily exert therapeutic effects by mimicking the activity of oxidoreductases. Among oxidoreductases, superoxide dismutase (SOD), catalase (CAT), and glutathione peroxidase (GPx) are the most typical and abundant metal protein antioxidants in nature. They play a critical role in inflammation treatment by scavenging ROS, regulating signaling pathways and metabolism, and promoting angiogenesis and tissue repair [[73], [74], [75]]. Peroxidase (POD) and oxidase (OXD) are classic pro-oxidative nanozymes that catalyze the conversion of H_2_O_2_ into reactive oxygen species. They can locally generate large amounts of ROS either through their inherent catalytic activity or in combination with therapies like photothermal and sonodynamic therapies, which can kill bacteria and prevent wound infection, offering significant potential for alleviating sustained inflammation.

#### 3.1.1 POD nanozyme

POD catalyzes the oxidation of various substrates by H_2_O_2_, having a dual role in detoxifying hydrogen peroxide and phenolic and amine compounds (such as formaldehyde and ethanol). POD has a heme cofactor at its catalytic site, where Fe(III) forms four coordination bonds with the porphyrin IX derivative [76]. The rate constant for the interaction of H_2_O_2_ is several orders of magnitude higher than that of the Fenton reaction [77]. Inspired by the metal ions in natural enzymes, Yan Xiyun's team first reported in 2007 that Fe_3_O_4_ has peroxidase-like enzyme activity, showing similar K_m_ values and higher k_cat_ and k_cat_/K_m_ values than natural horseradish peroxidase (HRP) [78]. Further studies found that Fe_3_O_4_ exhibits pH-dependent dual enzyme-like activity, showing POD activity under acidic conditions and CAT activity under neutral and alkaline conditions [79].

#### 3.1.2 OXD nanozyme

OXD catalyze oxygen reduction reaction (ORR)-like processes by promoting the oxidation of various substrates using oxygen (O_2_), generating H_2_O_2_, H_2_O, or ·O_2_^−^ [80]. Oxidases are enzymes that oxidize substrates directly with oxygen, and unlike traditional peroxidases, they do not require exogenous H_2_O_2_, instead using dissolved O_2_ to carry out oxidation reactions and generate ROS, contributing to antibacterial, antioxidant, and signaling functions [81]. The mechanisms of oxidases are often closely associated with the catalytic properties of metal ions or metal oxides. For example, metals or metal oxides like copper oxide (CuO) and Au can adsorb and activate O_2_, generating superoxide anions or singlet oxygen, which facilitates electron transfer. During this process, the metal's valence state cycles between M^0^/M^+^ or M^+^/M^2+^, avoiding the potential toxicity of exogenous oxygenating agents while generating ROS in situ, which enhances antibacterial activity.

#### 3.1.3 CAT nanozyme

CAT is one of the biological enzymes with the highest turnover rate (the rate at which substrates react): under saturated enzyme conditions, a single CAT enzyme can catalyze the dismutation of 40 million H_2_O_2_ molecules per second into H_2_O and O_2_, thereby protecting cells from the toxic effects of H_2_O_2_ [82]. CAT is predominantly expressed in peroxisomes of the liver, kidneys, mitochondria, and the cytoplasm of red blood cells, and it also plays a role in the growth of fibroblasts, melanoma cells, and mastocytoma cells [23,83]. CAT proteins can be divided into single-function heme-containing proteins, double-function heme catalase peroxidases, and manganese-containing CAT proteins [84].

CAT catalyzes the dismutation of H_2_O_2_ using the Fe atom in its heme cofactor as the catalytic site [85].Zhang et al. [86] developed a defective Fe-N_4_ single-atom nanozyme through edge-site engineering, which exhibited outstanding CAT-like activity (52.64 U/mg) at a relatively low iron loading of 0.45 %, surpassing natural CAT enzymes and demonstrating excellent potential for replacing natural enzymes in the treatment of inflammation.

#### 3.1.4 SOD nanozyme

SOD is the main enzyme that defends against ·O_2_^−^. It catalyzes the dismutation of ·O_2_^−^ into O_2_ and H_2_O, using free radicals as substrates. The reaction kinetics are diffusion-limited, approximately 10^9^ mol^−1^s^−1^ [87,88]. Based on the metal redox pairs involved, SOD can be classified into four types: Cu(II/I)-Zn(II), Fe(III/II), Mn(III/II), and Ni(III/II) [89]. Depending on their location, SOD is further divided in mammalian cells into cytosolic CuZn-SOD (SOD1), mitochondrial Mn-SOD (SOD2), and extracellular CuZn-SOD (SOD3/EC-SOD) [90]. The active site of natural CuZn-SOD consists of metal atoms Cu and Zn, where Cu acts as the catalytic site and Zn helps stabilize the enzyme [91]. Synthetic Cu has currently demonstrated excellent potential to replace natural SOD enzymes, with widespread and in-depth research.

#### 3.1.5 GPx nanozyme

GPx uses selenocysteine as the catalytic center and glutathione (GSH) or other related thiols as cofactors to decompose ROS (such as PODs) in cells into water or alcohol, effectively removing these harmful substances [75]. Additionally, GPx can also eliminate lipid and phospholipid radicals, protecting cell membrane structures and biomolecules (such as lipoproteins and DNA). In 1973, Flohé et al. [92] first confirmed through X-ray analysis that the selenium atom is located at the active site of GPx, indicating that selenium has enzyme-like activity potential. In 2014, Mugesh et al. [68] found Vanadium (V) as a metal active site in haloperoxidases, exhibiting GPx-like activity with its metal oxide V_2_O_5_ nanowires, emonstrating the potential of metal nanozymes in GPx-like enzyme activity.

#### 3.1.6 Cascade enzyme activity

In biochemical systems, cascade reactions refer to a series of enzyme-catalyzed reactions that occur in a predefined sequence: the product of one reaction becomes the substrate for the next, providing advantages such as high efficiency and minimal resource waste [93,94]. Nanozymes are not restricted to simulating the function of a single natural enzyme; rather, they can exhibit two or more complementary enzyme-like activities simultaneously, generating a cascade catalysis effect. For instance, many nanozymes with CAT activity also display SOD or POD activity, allowing them to clear ·O_2_^−^ and catalyze both the generation and decomposition of H_2_O_2_ within the same microenvironment, thus mimicking a natural cascade reaction without complex compartmentalization [85]. Inspired by the geometric shapes of human mitochondrial Mn-SOD and human erythrocyte CAT enzymes, Yang et al. constructed an ultra-thin 2D ZMTP nanosheet, which regulates the reversible conversion of Mn valence between +3 and + 5/+2 by introducing Zn with manganese porphyrins, achieving both strong SOD and CAT activities [95]. Lu et al. [96] developed the classic Prussian blue nanozyme (Fe^2+^-C ≡ N-Fe^3+^),which exhibits CAT, SOD, and POD activities, serving as a multi-functional ROS regulator. Li et al. [97] also found that MoS_2_ nanosheets (MoS_2_ NS) exhibit CAT, SOD, and POD activities, with further enhancement of its activities achieved through combined photothermal therapy.

### 3.2 Other regulatory mechanisms

Typically, nanozymes regulate ROS by modulating macrophage polarization (M1 to M2) at the cellular level [26], and at the molecular level, they inhibit inflammatory pathways [98], reduce pro-inflammatory cytokines, and upregulate antioxidant genes, thereby achieving precise anti-inflammatory effects [99,100].

In cellular inflammation and stress response mechanisms, MAPK and NF-κB signaling pathways serve as core regulatory hubs, whose aberrant activation is closely associated with various pathological processes. Recent studies have revealed that metallic nanozymes, as efficient exogenous intervention tools, can simultaneously regulate two or more signaling pathways. For instance, TPP-Au-Ru [101], Pt@PCN222-Mn [102], and cerium-based nanoparticles (CeO_2_ NPs) [103] have been demonstrated to synchronously inhibit the activation of both MAPK and NF-κB signaling cascades, thereby effectively reducing inflammatory factor release and improving mitochondrial function in models of inflammation and osteoarthritis. Beyond the co-regulation of these two core pathways, different nanozymes exhibit specific regulatory capacities for other signaling routes: LC-AuNCs can simultaneously influence the MAPK/ERK/NF-κB and PI3K-AKT pathways, guiding macrophages toward M2 phenotype polarization [104]; CuCo_2_O_4_ primarily targets the JAK-STAT and NF-κB signaling networks [105], demonstrating unique immunomodulatory functions; while CeO_2_@GNSs/Myr-HA specifically inhibits the p38/MAPK pathway, effectively suppressing angiogenesis [106]. These findings systematically reveal that multi-target intervention strategies based on metallic nanozymes can differentially regulate key signaling nodes, offering significant therapeutic potential in inflammatory diseases, immune-metabolic abnormalities, and microenvironment regulation.

Further investigation into their mechanisms of action shows that the preferential regulatory capacity of nanozymes toward signaling pathways primarily depends on their intrinsic physicochemical properties. Among these, the valence states of metal ions and interfacial electron transfer processes play decisive roles. The variable valence states of metal ions are core factors in regulating catalytic selectivity. For example, the antioxidant and anti-inflammatory efficacy of cerium-based nanozymes is directly attributed to the coexistence and rapid switching of Ce^3+^/Ce^4+^ redox pairs on their surface, enabling efficient scavenging of superoxide radicals and hydrogen peroxide, thereby effectively inhibiting the activation of the ROS-NF-κB/MAPK pathway and blocking NLRP3 inflammasome assembly and activation [107]. In contrast, the Fe^2+^/Fe^3+^ cycle in iron-based nanozymes drives the conversion of H_2_O_2_ into highly toxic ·OH, favoring the activation of pro-apoptotic and pyroptotic pathways to achieve antibacterial effects, rather than anti-inflammatory pathways [108]. Meanwhile, in metallic hybrid systems, electron interactions between different components can reshape catalytic activity and specificity. For instance, in alloy nanozymes such as Au-Pt [109], electron transfer from metals with lower work functions to those with higher work functions not only enhances catalytic efficiency but also finely tunes reaction pathways, enabling a preference for specific reactions in complex biological environments, thereby achieving differential regulation of distinct signaling pathways.

The ability of metallic nanozymes to simultaneously intervene in multiple signaling pathways, while offering synergistic therapeutic potential, also introduces significant complexity into rational design. This multi-target nature primarily stems from their inherent catalytic broad-spectrum nature, which may lead to the simultaneous generation or consumption of multiple reactive oxygen species (e.g., H_2_O_2_, ·OH, ·O_2_^−^) within cells. These signaling molecules can concurrently activate or inhibit different signaling networks. For example, a nanozyme possessing both peroxidase and oxidase activities might consume H_2_O_2_ to suppress one pathway while simultaneously generating superoxide to activate another. Additionally, the subcellular localization of nanozymes determines which signaling components they interact with; for instance, mitochondria-targeting nanozymes may more readily influence apoptosis pathways, while those localized in the cytoplasm may preferentially regulate MAPK or NF-κB pathways. Facing this complexity, research trends are shifting from the pursuit of single enzyme activities toward more refined precision targeting strategies. These include achieving organelle-specific targeting through surface functionalization, thereby transforming the unpredictability of multi-pathway intervention into programmable, synergistic therapeutic effects. An example is the TPP-Au-Ru nanozyme [101], which utilizes triphenylphosphonium-mediated mitochondrial targeting to concentrate its catalytic activity near mitochondria, thereby more efficiently protecting mitochondrial function and indirectly attenuating MAPK/NF-κB activation.

Beyond ROS and classical signaling regulation, nanozymes can also modulate metabolic processes involved in inflammation. The unique pathological microenvironment created by inflammation is often accompanied by the abnormal accumulation of metabolic products such as lipids and glucose. While hyperglycemia and hyperlipidemia contribute to excessive ROS production, impair angiogenesis, delay tissue repair, and create conditions conducive to bacterial colonization [110,111]. Nanozymes can intervene in related metabolic pathways to reverse these effects [112]. For example, gold nanozymes have demonstrated glucose oxidase-like activity, catalyzing the oxidation of glucose [113,114]. They also regulate multiple glucose metabolism pathways, including glycolysis/gluconeogenesis, pyruvate metabolism, PPAR signaling, and insulin signaling [115]. Additionally, they reduce the accumulation of lipid PODs in lipid metabolism [17]. Beyond directly regulating metabolic pathways, nanozymes can also modulate the gut microbiota and systemic metabolism by altering the expression of hepatic genes related to glucose and lipid metabolism, and by increasing the abundance of beneficial bacteria in feces—ultimately affecting both inflammation and metabolic homeostasis.

In addition to modulating the host response, nanozymes can exert antibacterial effects by directly disrupting bacterial metabolism, thereby indirectly controlling infection-associated inflammation. Their mechanisms of action include inhibiting bacterial ATP synthase to induce energy depletion [116], interfering with the metabolism of key amino acids such as glycine, arginine, and serine [117], and disrupting bacterial metal homeostasis or aromatic amino acid biosynthesis under acidic conditions [118].

## 4 Rational design of metallic hybrid nanozyme

To date, metallic nanozymes constitute the majority among the vast library of nanozymes developed by scientists. Metallic nanozymes refer to a class of enzyme-mimicking materials that exhibit catalytic functions at the nanoscale [119], with metal components existing in forms such as metallic elements [120], metal oxides [121], polyoxometalates (POMs) [122], or single atom nanozymes (SANs). These materials can be categorized into single-metal, bimetallic, and multi-metallic nanozymes, with several representative examples and their enzymatic activities summarized in Table 1.

**Table 1:** Enzyme-like activities of different metallic nanzymes

Class	Nanozymes	Enzyme-like Activity	Bibliography
Fe	Fe_3_O_4_	POD (Acidic Conditions) /CAT (Alkaline Condition)	[123]
	Fe-N-C	POD	[124]
Cu	Single-Cu-atom	POD	[125]
	Hollow CuS	POD、GPx	[126]
	Cu_*x*_O	POD、SOD、CAT、GPx	[127]
Mn	MnO_2_ Nanoflakes	POD	[128]
	MnO_2_ Nanoshells	SOD、CAT	[129]
	Mn_3_O_4_	SOD、CAT、GPx	[130]
Mo	MoO_3_	OXD	[131]
	MoS_2_	CAT、SOD、POD	[97,131]
Co	Co_3_O_4_	POD、CAT	[132]
Ce	CeO_2_	POD/CAT (Acidic Condition)	[133]
Au	Au	POD、CAT、SOD、GOD	[134,135]
Ag	Ag	CAT (Alkaline Condition) /POD (Acidic Condition)	[136]
Pt	Pt	、POD	[137]
Pd	Pd	SOD、CAT、POD	[138]
Rh	Rh	CAT	[139]
Ru	Ru	POD、GOx	[140]
Bimetallic hybrid nanozymes	Au-Pt	CAT、POD	[109]
	Au-Ru	POD	[141]
	CrFeO_3_	CAT	[142]
	Ru-TiO_2_	POD	[143]
	Pd-Fe_3_O_4_	POD	[144]
	Fe@MoS_2_	POD	[145]
	Pt@PCN222-Mn	SOD、CAT	[22]
Multimetallic hybrid nanozymes	Ir-Coated Pd-Pt	POD	[146]
	Pd-Pt-Ru	POD	[147]
	Fe_3_O_4_@MoS_2_-Ag	POD	[148]
	MnO_2_/Ce6@ZIF-8	CAT	[149]
	FeMo_6_ @Ce-UiO-66	POD、OXD	[150]
	MnO_2_@PtCo	CAT、OXD	[151]

### 4.1 Definition and classification of metallic hybrid nanozymes

Metallic hybrid nanozymes are defined as a class of high-performance catalytic materials constructed through the combination or integration of two or more distinct metallic components at the nanoscale [13]. The term "hybridization" in this context does not imply simple physical mixing, but rather refers to the formation of sophisticated structures—such as alloys, core-shell configurations, heterojunctions, or single/dual-atom hybrids—via specific synthesis strategies (e.g., hydrothermal methods, seed-mediated growth), which enable synergistically enhanced functionalities [152]. This concept distinguishes them from single-metal nanozymes, which often exhibit relatively singular catalytic functions and are subject to inherent limitations [153]. Metal-organic framework (MOF)-based materials have been extensively explored in the field of nanozymes [154,155]. Notably, when the enzyme-like activity of a MOF originates from the synergistic integration of its dual (or multi) metal nodes at the molecular level, the MOF itself can be considered an important structural form of metallic hybrid nanozyme [156,157].

Compared to single-metal nanozymes (such as monometallic MOFs, POMs) and simple physical mixtures of nanomaterials, these metallic hybrid nanozymes exhibit superior performance. Their distinct advantage lies in the precisely controlled composition and spatial arrangement of multiple metal centers within a single, stable framework. This unique structure enables synergistic catalytic effects that are unattainable in other systems, often leading to enhanced activity, superior substrate specificity, and the ability to mimic complex natural enzymatic cascades. For instance, Shen et al. [158] discovered that when combined with Au NPs and Pt NPs, the CAT activity of APHPB NPs was more than twice that of standalone HPB NPs, demonstrating enhanced enzyme-like activity. Wang et al. [159] discovered that Pd@TiO_2_ generated 6 mg L^−1^ oxygen in 45 s, while Pd NSs took 135 s to produce the same amount. When composite nanozymes are composed of enzymes with different catalytic functions, they can further improve enzyme activity or achieve better cascade catalysis. For example, CeO_2_ inherently exhibits both CAT and SOD activity. Wang et al. [107] enhanced its POD activity significantly by doping it with Cu and increasing the Ce^3+^/Ce^4+^ ratio. The RuCu NS synthesized by Yang displayed not only POD activity and ·OH generation but also SOD and GPx functions. ·O_2_^−^ was converted to H_2_O_2_ under the influence of SOD activity, which was further catalyzed at the POD site to generate more OH; at the same time, the nanosheets consumed GSH to prevent the elimination of ·OH [160].

Based on their structural design principles at the nanoscale, they can be primarily classified into the following four categories.

#### 4.1.1 Alloy-structured nanozymes

This type of nanozyme is synthesized using strategies such as chemical co-reduction methods, enabling different metal atoms to form solid solution structures through uniform mixing at the nanoscale (e.g. Au-Pt alloys [161]). Their catalytic properties originate from the electronic synergistic effects between different metal atoms: by precisely adjusting the metal composition ratio, the d-band center of the material can be effectively modulated, thereby optimizing the adsorption behavior of reaction intermediates and achieving simultaneous.

Hancement of catalytic activity and selectivity. In terms of ROS regulation, this unique capability for fine-tuning the electronic structure allows them to preferentially favor specific ROS conversion pathways in complex biological microenvironments, such as efficiently converting superoxide anions •O_2_^−^ into hydrogen peroxide H_2_O_2_.

#### 4.1.2 Core-shell structured nanozymes

Core-shell structured nanozymes (e.g. Au@Pt [162]) can be constructed using techniques such as seed-mediated growth. The catalytic advantages of these materials primarily stem from interfacial effects: utilizing lattice mismatch between different metals to generate strain effects, combined with electronic interactions at the interface, significantly enhances the catalytic activity and structural stability of the outer shell. This structural design enables precise functional (division of labor); for instance, the core can be used for medical imaging or photothermal conversion, while the shell is responsible for catalytically scavenging ROS, providing an ideal platform for theranostics.

#### 4.1.3 Heterojunction-structured nanozymes

Heterojunction-type nanozymes (e.g.CuO_2_/TiO_2_ [163]) can be constructed using hydrothermal/solvothermal methods. The core of their catalytic mechanism lies in the built-in electric field formed at the interface, which effectively promotes the separation of photogenerated electron-hole pairs, thereby greatly enhancing the efficiency of photocatalytic generation or consumption of ROS. This characteristic makes them particularly suitable for combination with photodynamic/sonodynamic therapy, enabling on-demand and precise regulation of ROS levels at the lesion site through external stimuli (light/ultrasound).

#### 4.1.4 Dual-atom hybrid nanozymes

Dual-atom hybrid nanozymes (e.g., Mn/Zn-DAN [164], FeMn-DAN [165]) can be prepared via processes such as pyrolysis or low-temperature self-assembly. These materials feature well-defined active centers and near 100 % atom utilization efficiency, exhibiting extremely high intrinsic catalytic activity. Their unique advantage lies in the ability of multiple adjacent atomic sites to synergistically catalyze multi-step ROS conversion reactions (e.g., superoxide dismutase reaction followed by catalase reaction), thereby achieving highly efficient cascade antioxidant effects.

**Fig. 4:**
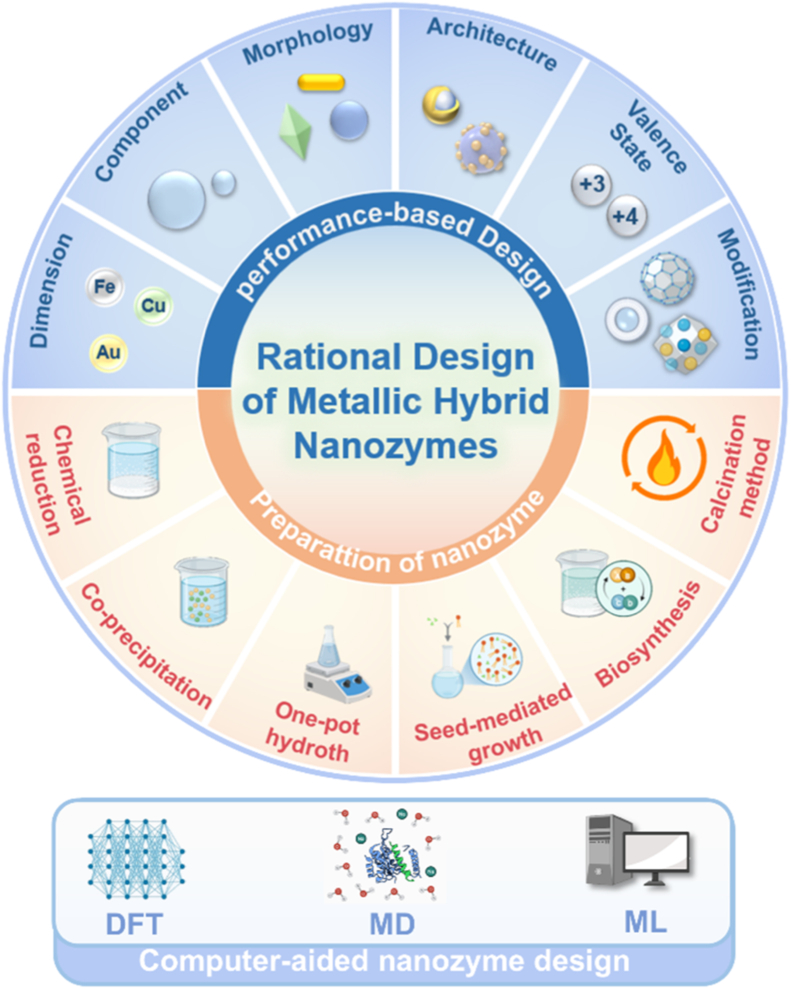
The rational design of metallic hybrid nanozymes enables the development of high-performance systems by integrating strategies like intelligent structural tuning, computational modeling, and precision synthesis. (Created with BioRender.com).

In summary, the core advantages of metallic hybrid nanozymes stem from their precise interfacial and electronic structure design. Through the four main hybridization strategies described above, it is possible not only to create more abundant and efficient active sites but also to integrate multiple catalytic functions and physical properties into a single entity. This multi-level structural design enables them to achieve more precise, efficient, and intelligent regulation of the ROS network in complex pathological microenvironments compared to single-metal nanozymes, through various mechanisms such as multi-metal synergy, electronic modulation, cascade reactions, and functional integration, providing a powerful technological platform for the treatment of inflammatory diseases (Fig. 4).

### 4.2 Synthesis method of metallic hybrid nanozymes

As aforementioned, in recent years, multi-metallic-based nanozymes have emerged as a promising class of catalysts with enhanced catalytic activity and selectivity for various applications including catalytic therapy [166]. Synthesis of multi-metallic-based nanozymes involves the careful selection and combination of different metal elements to create synergistic effects that can amplify the catalytic performance. However, the inherent thermodynamic instability of multi-metallic-based nanomaterials poses significant challenges in the development of novel synthesis methods for their preparation [167]. This section focuses on the key synthesis methods used in the fabrication of multi-metallic-based nanozymes, including the widely used traditional wet chemistry method and ultrafast synthetic strategies.

The synthesis of metal nanoparticles primarily encompasses top-down physical approaches, bottom-up chemical strategies, and other emerging techniques. In nanomaterial fabrication, physical methods typically involve reducing bulk metal sources into nanoscale particles through mechanical or physical means, such as ball milling, photolithography, and laser ablation. However, these methods often suffer from limitations in precise control over particle size and structure, and may introduce impurities during processing [168]. In contrast, bottom-up chemical synthesis has gained broader application in the fabrication of metallic hybrid nanomaterials, owing to its simplicity, high controllability, and minimal equipment requirements. Various studies have systematically reviewed the common physical and chemical methods used for nanoparticle synthesis [169]. This work will focus specifically on solution-based chemical synthesis strategies. Among these, the most widely adopted and well-established techniques include chemical reduction, thermal decomposition, one-pot synthesis, seed-mediated growth, co-precipitation, and hydrothermal methods [170]. These approaches offer exceptional tunability in morphology, size, and structure, providing a robust foundation for the controlled fabrication of functional nanomaterials (Table 2).

**Table 2:** Metallic hybrid nanozymes synthesized using different methods.

Synthesis method	Composition	Temperature (°C)	Required reagents	Bibliography
Chemical reduction method	Pt-Ag	Room temperature	NaBH_4_	[171]
Co-precipitation method	Mn-Ce	400 °C	Ammonia	[172]
One-pot hydrothermal synthesis	Pd-Zn	120 °C	/	[173]
	Cu -Mn	90 °C	Benzoic acid、DMF	[174]
	Ni-Pt	280 °C	Acetaldehyde/TEG	[175]
	Pt-Sn	50 °C–80 °C	NaBH_4_	[176]
	Au-Pt-Ni	60 °C	AA	[177]
Seed-mediated growth	Ni-Pt	600–800 °C	/	[178]
Biosynthesis	Ag-Cu	70 °C	Toddy	[179]
	Cu-Zn	70 °C	Toddy	[179]
	Au-Ag	Room temperature	CB extract	[180]
Calcination method	Zn-Cu	400 °C	/	[181]

#### 4.2.1 Chemical reduction

Chemical reduction is one of the most straightforward and widely adopted wet-chemical methods for synthesizing nanomaterials. In this approach, metal ions (M^n+^) are reduced to their elemental state (M^0^) by a reducing agent, leading to the formation of nanoparticle clusters in solution. Metal precursors are typically provided as soluble metal salts or as metal complexes formed by coordination between metal cations and appropriate ligands. The selection of reducing agent plays a critical role in determining the size, morphology, dispersity, and composition of the resulting nanoparticles. Strong reducing agents such as NaBH_4_ are frequently employed for the rapid synthesis of monodisperse metallic nanoparticles (e.g., Au-Ag, Au-Pt, Cu-Au alloys). For instance, Jiang et al. [182] utilized NaBH_4_ to incorporate AuCu alloy catalysts into TiO_2_ nanosheets, resulting in a composite material with superior photocatalytic activity. However, the high reduction rate induced by strong reducing agents can lead to uncontrolled growth of large particles, posing challenges for precise nanoscale synthesis.

By contrast, milder reducing agents—such as sodium citrate, ascorbic acid, ethylene glycol, and oleylamine—provide improved kinetic control and enable fine regulation of nanoparticle formation. These agents often reduce the metal ion with the higher redox potential first, forming a metallic core, followed by reduction of the second metal onto the core surface, yielding core-shell structured nanoparticles. For example, Fen et al. [183] employed ascorbic acid to rapidly and conveniently synthesize core-shell Au@PtRu nanorods. Similarly, Tan et al. [184] used hydrazine hydrate to effectively deposit Ag/Pd bimetallic nanoparticles onto TiO_2_ nanosheets, thereby enhancing their catalytic efficiency.

In addition, mixed reducing agent systems have also been widely applied in nanoparticle synthesis. For instance, Liu et al. [185] used both NaBH_4_ and ethylene glycol as co-reducing agents to synthesize Pd/Li-decorated silicon-based nanosheets through a topological reaction. This mixed reductant strategy provides better control over the reaction process, thereby improving the uniformity and tunability of the resulting nanoparticles.

#### 4.2.2 Co-precipitation

The co-precipitation method is one of the earliest wet-chemical routes used for preparing inorganic nanoparticles. Its core principle is to allow multiple metal ions to simultaneously reach supersaturation and co-precipitate in the same reaction system, thereby achieving homogeneous elemental mixing at the molecular scale. A typical procedure involves dissolving metal salts in water or other polar solvents, followed by the slow addition of precipitating agents such as OH^−^ or CO_3_^2−^. The nucleation rate is governed by reaction temperature, pH, and ionic strength, while capping agents like sodium citrate, PEG, and PVP suppress particle agglomeration during the aging and calcination stages via electrostatic or steric hindrance. Functional properties such as magnetism, biocompatibility, or catalytic activity can be introduced through subsequent calcination or surface modification. These capping agents also serve as forward-looking strategies for preventing nanoparticle aggregation, thereby enhancing the colloidal stability of the product [186]. Amit A. Vernekar et al. [187] synthesized MnFe_2_O_4_ nanoparticles by using a straightforward co-precipitation approach, and demonstrated that changes in particle morphology markedly influenced their nanozyme catalytic activity. Similarly, Kim et al. [188] employed NH_4_OH as the precipitating agent to achieve size-tunable MnFe_2_O_4_ nanoparticles (6–12 nm).

#### 4.2.3 One-pot hydrothermal synthesis and thermal decomposition

The one-pot hydrothermal synthesis method has gained increasing attention in recent years due to its simplicity, high yield, and low cost. However, due to the absence of purification steps, this method carries a relatively higher risk of side reactions. Therefore, selecting highly selective catalysts and optimizing reaction conditions are key to ensuring the success of the one-pot synthesis approach. Hao et al. [189] successfully synthesized core-shell Au-M@SiO_2_ (M = Rh, Pd, Ir, Pt) nanoparticles using a one-pot method, and the enhanced catalytic activity resulting from the bimetallic synergistic effect further demonstrated the wide applicability of this approach in nanoparticle synthesis.

In addition, thermal decomposition in non-aqueous media by introducing organometallic or noble metal salt mixtures under an inert atmosphere is also a commonly used and effective method for synthesizing noble metal-based composites. Compared with aqueous-phase synthesis, thermal decomposition offers narrower particle size distributions and better compositional tunability. Noble metal nanostructures can be combined with many transition metals that are not compatible with aqueous synthesis, thereby expanding their application scope. This method is not only easy to operate and cost-effective but also provides better control over particle size. However, it also has obvious drawbacks: reducing agents can produce self-nucleated byproducts, and additional purification and post-treatment steps are required to obtain the desired nanoparticles. Nevertheless, thermal decomposition remains one of the classic methods for synthesizing MnFe_2_O_4_ [190,191]. Asghar et al. [192] found that MnFe_2_O_4_ nanoparticles synthesized via thermal decomposition had smaller grain sizes (5 nm) compared to those obtained by co-precipitation (9.5 nm).

#### 4.2.4 Seed-mediated growth

The structural feature of alloy nanoparticles is the spatially uniform distribution of two or more metal elements in the form of a statistical mixture. When different metal precursors are present simultaneously, co-reduction can be used for synthesis [193]. During synthesis, when different metal precursors are spatially separated, sequential reduction (seed-mediated growth) can lead to the formation of core-shell, satellite, and Janus nanostructures [194].

Seed-mediated growth has been demonstrated as one of the most effective strategies for synthesizing NMBNPs and Janus structures. This method involves nucleation and growth of a second metal at specific sites on pre-synthesized monometallic seeds with defined structures, making it suitable for constructing heterometallic and multimetallic nanomaterials. The process includes two steps: reduction of the metal precursor and deposition of metal atoms onto the existing seed. Jessi et al. [195] used Au nanorods as seeds, epitaxially grew Pd, and then coated them with mesoporous SiO_2_ to fabricate Au@Pd@SiO_2_ core-shell nanorods. The mesoporous layer stabilized the overall structure while exposing active sites, leading to a ∼50-fold improvement in catalytic activity compared to alloy or monometallic counterparts. In a similar material system, Zhang et al. [196] systematically evaluated the size-performance relationship of Au@Pd core-shell catalysts. They found that the methanol oxidation activity was linearly correlated with the core diameter of Au and non-linearly related to the Pd shell thickness, providing a quantitative basis for the precise design of such nanozymes. For improved synthetic control, Muzzi et al. [197] introduced oleylamine as a dynamic surfactant that undergoes reversible adsorption and desorption on the metal surface, forming a dynamic interfacial layer. This approach enables precise control over the growth of nanoparticles as well as the nucleation of a secondary inorganic phase, thereby enhancing the uniformity and yield of multimetallic or multiphase nanostructures.

When the synthetic goal shifts toward highly asymmetric Janus structures, the high geometric symmetry and equivalent surface sites of seed particles make it easy for the second metal to nucleate and grow in random positions, leading to the formation of core-shell or satellite structures instead of true Janus structures. Therefore, breaking the symmetry of the second metal and promoting anisotropic growth is essential. This can be achieved by tuning reduction kinetics, choosing appropriate capping agents, and controlling lattice mismatch between seed and deposited metals. Li et al. [198] proposed a hydrophilicity-mediated interfacial selective assembly strategy: using an asymmetric template composed of hydrophilic SiO_2_ and hydrophobic PMO domains, metal compounds selectively deposit on the SiO_2_ subunit. This strategy enabled the construction of various M-mJNPs, and their structure could be tuned by adjusting the shape and position of the hydrophilic region within the template. In the context of nanozyme self-assembly, Qu et al. [151] first synthesized PtCo nanoparticles as active seeds and then induced the self-assembly of MnO_2_. By adjusting the precursor ratios, they successfully obtained highly ordered MnO_2_@PtCo nanoflowers, which showed excellent catalytic efficiency in intracellular mimic reactions.

#### 4.2.5 Green biosynthesis

In addition to traditional chemical and physical methods, emerging green technologies such as biosynthesis and rapid self-assembly have recently provided new strategies for nanozyme fabrication. Biosynthesis, which utilizes plant or microbial extracts as both reducing and capping agents, features mild reaction conditions and eco-friendly processes, making it a widely favored synthesis method [199]. For example, in addition to their genetic roles, DNA and nucleic acid templates serve as interesting ligands for various metal ions. Inspired by such coordination chemistry, Du et al. [200] employed a coordination-driven one-step rapid self-assembly method to synthesize Ag@Pt nanozymes. In this strategy, single-stranded DNA was used as a template, and by mixing precursor materials (AgNO_3_ and K_2_PtCl_4_) with the DNA at 95 °C for 4 min, Ag@Pt nanozymes with tunable sizes were successfully produced. Similarly, Yang et al. [108] constructed amorphous/crystalline heterostructured Fe-DNA nanozymes using the same concept, demonstrating that DNA can directly coordinate with Fe^2+^/Fe^3+^ ions and drive the self-assembly process without the need for additional reducing or stabilizing agents. With the same goal of achieving sustainable and cost-efficient nanozyme production, Qamar et al. [201] introduced a biosynthesis strategy using plant extracts, bacteria, and fungi as integrated sources of reducing and capping agents. This green method employs natural substances that simultaneously act as capping, stabilizing, and reducing agents.

Compared to traditional chemical methods, green technologies for nanoparticle synthesis are more cost-effective, less toxic, and facilitate large-scale production using sustainable raw materials, with successful applications in the synthesis of various nanozymes [202]. However, green synthesis faces several challenges, particularly its reliance on specific plant or microbial extracts, which may lead to fluctuations in raw material costs or supply instability, thus affecting the feasibility of large-scale production [203]. Additionally, a lack of comprehensive toxicological evaluations necessitates further preclinical and clinical trials to verify safety. Green synthesis also encounters issues related to storage stability and consistency of quality in industrial applications [204]. Therefore, while it holds significant potential for sustainable development, further research and improvements are needed in scaling up production and assessing biosafety.

This section has systematically reviewed the primary synthesis methods for metallic hybrid nanozymes. Each method possesses distinct characteristics:Chemical reduction and co-precipitation are classic, widely adopted in laboratories due to their simple operation and low cost, yet the former requires higher precision in reaction control, while the latter is more sensitive to reaction conditions like pH and temperature. One-pot hydrothermal synthesis offers high yield and a straightforward process but carries a relatively higher risk of side reactions. Seed-mediated growth demonstrates unparalleled advantages in precisely controlling nanostructures (e.g., core-shell, Janus), making it the preferred choice for designing complex architectures; however, its process is the most complex and demands extremely strict condition control. The emerging biosynthesis approach adheres to green chemistry principles and operates under mild conditions, yet it still faces challenges in controlling the uniformity of particle morphology and size, and scalability remains difficult. The conventional calcination method is primarily used for obtaining metal oxide nanozymes with high crystallinity. In summary, the selection of a synthesis method requires a comprehensive trade-off among reaction conditions, cost, product performance (size, morphology, structural complexity), and scalability potential. Future developments will likely lean towards combining multiple methods and leveraging computer-aided design to achieve precise control over the properties of metallic hybrid nanozymes.

### 4.3 Regulation strategy of metallic hybrid nanozymes

Broad consensus suggests that the catalytic activity of artificial metal nanozymes stems mainly from two mechanisms: one involves enzyme-like or redox-active catalytic centers [205], while the other arises from nanoscale effects such as surface phenomena, quantum size effects, and bulk effects. In contrast to natural metalloproteins, artificial metal nanozymes largely rely on their distinct surface characteristics for catalytic activity. Each fundamental step of surface catalysis—including substrate adsorption, diffusion, reaction, and desorption—is affected by surface engineering [206]. Hence, tailoring surface or internal geometric-electronic structures via compositional control, morphology engineering, valence state tuning, or external stimuli (e.g., light, ultrasound, magnetic fields) has become an essential strategy to boost catalytic efficiency [207].

A wide range of strategies can be employed in the design of metallic hybrid nanozymes, including tuning their chemical composition, structural architecture, surface properties, synthesis approaches, and post-treatment protocols. These approaches can be used alone or synergistically to enhance catalytic efficiency and broaden functional utility. Beyond conventional empirical synthesis, rational top-down design—guided by high-throughput computing and machine learning-based structure-function analysis—is gaining traction. This involves building primary frameworks followed by fine-tuned surface/interface engineering, ultimately yielding nanozymes with enhanced activity, lower detection thresholds, and improved therapeutic outcomes [208].

#### 4.3.1 Chemical composition

In bimetallic or multimetallic systems, the composition and ratio of constituent metals are central to generating synergistic effects [209]. Controlled doping of secondary metals can modulate the original metal's electronic state and geometry, enhance electron transfer, and increase the density of active sites [210]. When metal-metal interactions induce significant geometric reconstruction and charge redistribution, improvements in catalytic activity, selectivity, and durability can surpass those of individual components [211]. For example, introducing Zn into CuO nanoparticles enhances POD-like activity [212]; embedding Pt nanoparticles into a PCN-222-Mn framework imparts additional CAT functionality, achieving cascade catalysis [22]. Similar synergistic enhancements are reported in PtNi hollow nanospheres [213] and PtRu nanoclusters [214]. Indeed, while such synergistic effects are well-documented in catalytic applications, their specific implications in inflammatory environments warrant deeper exploration. A compelling illustration is provided by the systematic screening of metal-doped Prussian blue nanozymes (PBzymes), where incorporation of eight different metals (Fe, Co, Ni, Cu, Zn, Mn, Ce, Ru) yielded distinct enzymatic and photothermal profiles [215]. Notably, Ru-PBzyme emerged with superior comprehensive antioxidant activity, excelling in CAT-, SOD-like functions and reactive nitrogen species scavenging, while Fe-PBzyme and Ru-PBzyme demonstrated optimal mild photothermal performance. This underscores that even within an identical Prussian blue framework, the choice of metal critically dictates the nanozyme's functional emphasis.

The type of metal fundamentally determines the catalytic nature of a nanozyme—different metals possess distinct electronic structures and redox properties, which directly affect the reaction pathway and active sites. For instance, copper nanoparticles typically exhibit POD and OXD activities, while CeO_2_ inherently shows CAT and SOD activities. In addition, environmental conditions such as pH and temperature can modulate catalytic behavior. For example, Fe_3_O_4_ demonstrates POD-like activity under acidic conditions and switches to CAT-like activity in neutral environments [123]; similarly, silver nanoparticles show CAT-like activity in alkaline media and POD-like activity under acidic conditions [136]. The pH dependence of nanozyme activity may be attributed to shifts in the oxidation state distribution of metal ions under varying pH levels, which in turn alters their redox behavior and catalytic performance. Motivated by this, scientists have turned to valence engineering as a means of tuning nanozyme activity. Transition metals such as Mo [216], Ru [217], can exist in mixed valence states, each exhibiting different catalytic behavior. Adjusting the oxidation state distribution allows for fine control over enzyme-mimetic properties [71]. For example, the redox cycling of ceria (Ce^4+^ ↔ Ce^3+^) driven by oxygen vacancies enables tuning of catalytic function: high Ce^3+^ content enhances SOD-like activity, while lower Ce^3+^ levels favor CAT-like function [218]. Likewise, increasing Mn^3+^/Mn^2+^ ratios in Mn_3_O_4_ improves both CAT and GPx-like activities, with negligible impact on SOD-like behavior [130]. Wang et al. [219] used ZnMn_2_O_4_ (ZM) as a model to apply a valence engineering strategy, specifically investigating the effect of Mn valence at octahedral sites. They discovered a positive correlation between antioxidant activity and Mn^4+^ content. By systematically synthesizing a series of samples with increasing Li proportions (ZM, Li-2, Li-4, Li-6, and LM (LiMn_2_O_4_)), they increased the average Mn valence from +3 to +4. Consequently, the nanozymes' SOD-like activity exhibited a progressive enhancement, following the trend ZM < Li-2 < Li-4 < Li-6 < LM. Notably, while the initial ZM possessed only SOD-like activity, the incorporation of sufficient Li successfully endowed the resulting LM with integrated CAT- and GPx-like activities, transforming it into a self-cascading nanozyme. This precise manipulation highlights how controlled metal composition directly dictates therapeutic efficacy in an inflammatory context.

In bimetallic or multimetallic nanozyme systems, the composition and ratio of constituent metals fundamentally govern their catalytic properties and therapeutic performance. Different metal combinations generate distinct enzymatic activity profiles—such as preferential SOD/CAT-like activities for broad-spectrum antioxidant effects or enhanced POD/OXD-like functions for bactericidal applications—which determine their suitability for specific inflammatory disease contexts. The precise control of metal ratios enables fine-tuning of these catalytic behaviors, allowing optimization for particular pathological microenvironments. For instance, systems with balanced multi-metallic compositions often demonstrate superior efficacy in complex inflammatory conditions like rheumatoid arthritis or neuroinflammation, where coordinated anti-oxidative and immunomodulatory actions are required.

#### 4.3.2 Structural characteristics

Metal-based nanomaterials allow precise tuning of morphology, crystal structure, and size at the nanoscale, offering a high degree of freedom for the rational design of catalytic properties [220]. The geometric shape of nanozymes (e.g., spheres, cubes, nanosheets, nanowires) influences the exposure of active sites and substrate-binding affinity. For instance, 2D nanosheet structures often exhibit higher catalytic activity due to their larger specific surface area and abundant active sites.

Further studies have revealed that specific morphologies and structural evolution of materials also determine the type and strength of their enzyme-mimetic activity. For example, MnO_2_ in nanosheet form mainly shows POD-like activity, but when it transforms into a nanocapsule structure, it shifts toward SOD and CAT mimicry. Similarly, flower-like Mn_3_O_4_ nanostructures exhibit approximately 50–60 % higher SOD activity compared to their cubic, polyhedral, and hexagonal counterparts [130]. The CAT-like activity of Co_3_O_4_ follows the order: nanosheets > nanorods > nanocubes [221,222]. For iron oxide nanostructures, POD-like activity decreases in the order: nanoclusters > triangular plates > octahedra [223]. Fu and colleagues conducted further comparisons of morphological influence and reported that the POD activity ranked as follows: nanoclusters > nanoflowers > nanocubes [224]. Furthermore, the crystallographic properties of metal or metal oxide nanozymes, including surface facets and lattice imperfections, play a key role in defining catalytic performance. High-index facets generally have more unsaturated surface atoms, and lattice defects introduce new active sites, both of which can modulate electronic structure and enhance catalytic efficiency [225]. The size of metal nanozymes is another critical factor affecting their performance. In general, smaller particles offer a higher surface area-to-volume ratio, more substrate-binding sites, and greater catalytic activity. For example, smaller Fe_3_O_4_ and Au nanoparticles demonstrate significantly higher POD-like activity than their larger counterparts [78,226]. Moreover, ultrasmall particles are advantageous for crossing physiological barriers and achieving site-specific accumulation [227]. However, if the size becomes too small, van der Waals forces may cause aggregation, which could diminish overall catalytic activity.

Metallic hybrid nanozymes significantly enhance catalytic efficiency and specificity by integrating multiple metal species or multivalent ions, which synergistically regulate electronic structures and diversify catalytic centers. Typically, noble metal-based bimetallic nanoparticles (NMBNPs) fall into four representative structural categories: alloy, core-shell, core-satellite, and Janus structures. In alloy structures, two metal atoms are uniformly mixed at the nanoscale to form a solid solution. By tuning the metal ratio, the d-band center can be modulated, affecting intermediate adsorption and reaction pathways—typical systems include Pt-Pt and Au-Pt [228]. Core-shell structures consist of a metallic core coated with another metal shell, such as Au@Pt [162]. These configurations can leverage strain effects and interfacial electronic interactions to tune shell activity, improving both structural stability and catalytic selectivity [229]. Core-satellite structures are composed of a large central core surrounded by multiple small satellite particles. This design promotes enhanced electron coupling, increases active site density, and supports synergistic effects like photothermal or plasmonic enhancement. Janus-type bimetallic nanozymes exhibit asymmetric architecture, where distinct catalytic regions coexist on one particle, offering controlled spatial separation of reactions and advanced stimulus-responsive or multi-enzymatic functionalities [230].

Beyond bimetallic systems, trimetallic nanoparticles are emerging as promising candidates for high-performance nanozymes due to their complex electronic tunability and multiple synergistic effects. Structurally comparable to bimetallic nanozymes, trimetallic nanoparticles offer five main configurations: uniform alloy types with homogeneous metal mixing [231]; gradient-regulated core-shell-shell architectures [228]; core-shell-alloy systems combining interfacial synergy; spatially asymmetric Janus or polyhedral heterostructures [232]; and core-satellite models that promote charge transfer and hierarchical catalysis [233]. These structural strategies offer a new design paradigm for developing multifunctional, stimuli-responsive, and highly adaptable nanozyme systems with broad application prospects.

#### 4.3.3 Surface properties

Surface modification, which involves grafting specific ligands or molecules onto the nanozyme surface, enables regulation of surface charge, hydrophilicity/hydrophobicity, and substrate adsorption capacity. For instance, hydrophilic ligands such as polyethylene glycol (PEG), dopamine, chitosan, or zwitterionic polymers can form a dense hydration layer on the surface, reducing serum protein adsorption and prolonging circulation half-life. Additionally, outer-layer incorporation of targeting moieties (e.g., RGD peptides, folic acid, or antibody fragments) significantly enhances active accumulation at diseased sites. Hydrophobic or amphiphilic molecules can facilitate membrane permeation via hydrophobic interactions, while positively charged moieties like quaternary ammonium salts or peptide sequences improve binding to negatively charged cell membranes, thereby increasing cellular uptake.

Complementary to this, surface defect engineering enables further tuning of electronic structures and activation of additional catalytic sites by deliberately introducing oxygen vacancies, metal vacancies, or coordinatively unsaturated edge sites within the crystal lattice. In metal oxides such as CeO_2-x_, Fe_3_O_4-x_, and TiO_2-x_, oxygen vacancies can reduce metal-oxygen bond energy, increase surface electron density, and accelerate the conversion of H_2_O_2_ to •OH, thereby enhancing POD activity. Similarly, metal vacancies in MnO_2_ or Co_3_O_4_ expose more unsaturated metal sites, boosting adsorption and transformation of superoxide or hydrogen peroxide, thus enabling SOD/CAT-like synergism [234].

Recent studies reveal that oxygen vacancies not only boost catalytic activity but also significantly influence catalytic selectivity. For example, in ordered CeO_x_ nanowire arrays, oxygen vacancies regulate hot electron generation and modulate reaction pathways by strengthening electronic coupling between surface active sites and reactants, thereby improving both selectivity and energy efficiency [235]. Additionally, these vacancies enhance the efficiency of photocatalytic water splitting and CO_2_ reduction [236]. The methods for creating defects have expanded to include hydrogen or ammonia reduction, plasma treatment, UV or laser irradiation, and non-metal doping with S, N, or P to generate vacancies. Moreover, oxygen vacancies in certain metal oxide catalysts promote hydrogen molecule dissociation and spillover, thereby accelerating hydrogenation reactions. This underscores their broad application potential in thermal and photo-thermal catalytic systems [237].

#### 4.3.4 Preparation method

Different synthesis methods—such as chemical reduction, sol-gel, and hydrothermal techniques—significantly influence the structure and catalytic performance of nanozymes. For example, the hydrothermal method effectively suppresses particle aggregation and promotes precursor crystallization, often yielding highly dispersed particles with exposed crystal facets, thereby enhancing catalytic activity. In contrast, the sol-gel method enables the formation of three-dimensional interconnected porous structures at relatively low temperatures, making it particularly suitable for producing nanozymes with large specific surface areas [238]. Post-synthesis treatments—such as calcination or reduction—can also affect the catalytic activity of nanozymes. For instance, appropriate calcination helps remove organic impurities and enhances structural stability, thereby improving catalytic durability.

### 4.4 Computer-aided nanozyme design

The advancement of computer technology has led to its growing application in the field of pharmacy [41]. Computer-aided nanozyme design encompasses all computational techniques including quantum chemistry, molecular dynamics (MD), and machine learning (ML). With rapid advances in computational technologies, these methods are playing an increasingly important role in nanozyme design.

#### 4.4.1 Theoretical and kinetic simulations for mechanism elucidation

Density functional theory (DFT) calculations are especially valuable for revealing the microscopic mechanisms and kinetics of catalytic reactions at the atomic level. DFT enables atomic-level modeling of catalytic processes and the establishment of theoretical models for catalytic activity, offering a powerful theoretical tool for nanozyme design [10]. Gao et al. [239] proposed a general computational strategy for screening and designing POD-like nanozymes to predict their catalytic activity. Using iron oxide nanozymes as a model system, they constructed 15 surfaces with various chemical compositions, lattice defects, exposed crystal facets, and chemical modifications. The study investigated the molecular mechanisms and reaction kinetics of H_2_O_2_ oxidation by these surfaces and summarized a three-step catalytic pathway for the POD-like reaction. This work demonstrated that high-throughput computational screening can accelerate nanozyme discovery and provide experimental guidance. By combining DFT calculations, Gao et al. [240] systematically investigated the thermodynamic and kinetic characteristics of nanozyme catalytic processes and established two core principles—the electronic energy level principle and the adsorption energy principle—for guiding activity screening. These principles can be readily translated into computable algorithms to efficiently identify nanomaterials with intrinsic SOD-like activity. In a separate study, Li et al. [241] theoretically explored the reaction pathway of Au/Cu Hop nanoparticles in the catalytic oxidation of phenolic contaminants. Based on DFT calculations, they constructed a Cu-S cluster model to simulate the complete reaction process from hydroquinone adsorption to the final conversion into benzoquinone. The study revealed that although the reaction involves several endothermic intermediate steps, the overall process is thermodynamically feasible and exothermic. Furthermore, experimental validation using a fluorescent probe confirmed the generation of ·OH, supporting the proposed mechanism in which Au/Cu Hop nanozymes catalyze H_2_O_2_ to produce ·OH, thereby oxidizing ABTS via a POD-like catalytic pathway.

**Fig. 5:**
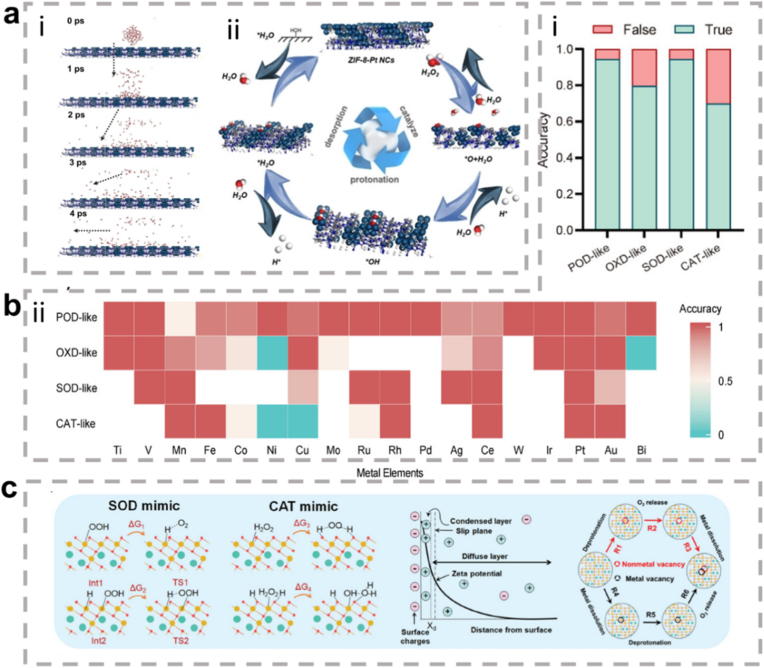
(a) (i) Dynamics processes of hydrogen peroxide-containing hydrospheres on monolayer cells of ZIF-8-pPt within 4 ps based on the COMPASS force field. (ii) Schematic representation of the optimal adsorption conformation and catalytic reaction cycle of ZIF-8-pPt for different adsorbents [243]. (b) (i) Prediction accuracy of the enzyme-mimicking types by inputting independent variables of the validation dataset into the model. (ii) Heatmap images of the prediction accuracy of the model by analyzing the enzyme-like activities of various transition metal elements [244].(c) Output information of AB2X4 database containing SOD barrier, CAT barrier, zeta potential, and acid stability[245].

MD is a computer simulation method that models the movement of atoms and molecules over time. By applying Newton's laws of motion, it predicts how a system of particles evolves, providing atomic-level insight into the behavior of materials and biomolecules [242]. DFT and MD combined simulations have been widely applied in nanozyme research. Yu et al. [243] systematically investigated the catalytic behavior of ZIF-8-pPt nanozymes using DFT and MD simulations. The unique electric field distribution and substrate channels of this structure significantly enhance the adsorption and decomposition of H_2_O_2_ (Fig. 5a). The synergistic effect between Pt nanoclusters and the microporous environment facilitates the heterogeneous cleavage of H_2_O_2_, efficiently generating highly reactive ·OH radicals. This process not only improves the catalytic efficiency but also provides a theoretical foundation for understanding the underlying catalytic mechanism. In summary, the integration of DFT and MD simulations has proven to be a powerful approach in nanozyme research. It successfully unravels key mechanistic insights, such as the enhanced reactive radical generation in confined microporous structures and the detailed thermodynamically feasible reaction pathways, thereby providing a fundamental theoretical basis for the observed high catalytic efficiency.

#### 4.4.2 Data-driven machine learning for performance prediction

ML, a branch of artificial intelligence, enables computers to autonomously discern patterns and relationships from data. In the context of nanozyme design, it facilitates the rapid construction of complex models that correlate material characteristics—such as composition and structure—with catalytic performance by analyzing existing experimental datasets. Leveraging these models, researchers can perform large-scale virtual screening of thousands of candidate materials and accurately predict their functionalities. This approach significantly expedites the research and development process, overcoming the inefficiencies and uncertainties inherent in traditional trial-and-error methods, and ultimately enables the efficient and rational design and optimization of nanozymes.

Vinogradov et al. established a fundamental framework for nanozyme development and prediction using ML [246]. The framework includes the analysis and classification of natural enzyme activities, data acquisition and management, ML algorithm development, prediction modeling, database scalability, and visualization tools. Their study analyzed data from over 100 published papers to explore the catalytic characteristics of nanozymes, providing a new approach for systematically and objectively evaluating nanozyme activity. Huang et al. [244] employed machine learning algorithms to elucidate the structure-performance relationship of nanozymes, enabling the classification and quantitative prediction of their enzyme-like activities. The model achieved prediction accuracies of 94.5 %, 79.7 %, 94.4 %, and 70.0 % for POD-, OXD-, SOD-, and CAT-like activities, respectively (Fig. 5b). Notably, its predictive capability for POD-like activity was the most outstanding, which can be attributed to the higher proportion of relevant samples in the training dataset. Sensitivity analysis further revealed that metal composition serves as a critical determinant of the enzyme-mimetic performance. The study also involved the synthesis of various ferritin (FTn)-based nanozymes incorporating single or hybrid metal elements. By quantifying protein and metal content and inputting these parameters into the model, the enzyme-like activity levels were successfully predicted. A strong agreement between the experimentally observed values and model predictions was obtained, validating both the analytical approach and the robustness of the model. It should be noted, however, that quantitative prediction of SOD- and CAT-like activities remains challenging due to the limited availability of relevant experimental data in the literature.

As artificial intelligence (AI) continues to advance, its integration into automated and intelligent nanozyme synthesis is opening exciting opportunities for rapid, high-efficiency material discovery [247].In recent years, the convergence of AI and ML with computational chemistry has sparked a new era of predictive nanozyme design, enabling unprecedented precision and throughput. Given the compositional complexity of nanomaterials and the ambiguous nature of catalytic sites, understanding structure-function relationships remains a central challenge in nanozyme research [248]. Conventional design approaches often depend on empirical knowledge and high-throughput trial-and-error, requiring extensive experimentation and significant time, cost, and labor. With a solid foundation in fundamental science, AI-assisted design can leverage ML algorithms to predict optimal synthetic pathways and material characteristics [249]. This data-driven strategy minimizes redundant experimentation, shortens development timelines, and shows vast promise in revolutionizing nanozyme discovery and precision material engineering. Kan et al. [250]demonstrated that by optimizing prompt engineering techniques for ChatGPT, the model can effectively assist researchers in efficient data collection and facilitate the development of an open-access web resource named AI-ZYMES for predicting the catalytic types and activities of nanozymes. Additionally, they constructed a ChatGPT-based nanozyme robot to guide laboratory synthesis procedures. This study illustrates the potential of ChatGPT to transform how researchers interact with the scientific literature and offers innovative application pathways for nanozyme research through the integration of ChatGPT with machine learning methodologies (Fig. 5c).

ML-assisted strategies are increasingly integrating into cutting-edge scientific research, offering a novel paradigm for new material development [251]. By combining machine learning with high-throughput screening, Yang et al. [245] successfully identified a multifunctional nanozyme, SrDy_2_O_4_, from the compositionally diverse AB_2_X_4_-type spinel materials for the treatment of ulcerative colitis (UC). The study first employed first-principles calculations to systematically screen element combinations at the A and B sites, extracting key electronic features such as orbital energy levels and electron density through wavefunction analysis to construct a high-quality material database. Furthermore, by integrating machine learning with the SISSO method, novel structural descriptors were developed that correlate material structural features with performance indicators including SOD-like activity, CAT-like activity, acid stability, and zeta potential (Fig. 5c). This approach revealed a semi-quantitative structure-activity relationship, enabling accurate and rapid prediction of target nanozymes. Based on the interactions between nanozymes and aminoglycoside antibiotics, Li et al. [252] developed a tri-channel nanozyme sensor array integrated with machine learning algorithms, enabling precise quantitative analysis of multiple aminoglycoside antibiotics in mixed samples. This sensor system effectively discriminates five distinct antibiotics without relying on concentration information and achieves accurate identification of complex mixed samples. Furthermore, the incorporation of deep neural network algorithms has led to significant breakthroughs in simultaneous quantification of multiple components, demonstrating exceptional analytical performance and application potential. In summary,by leveraging big data and machine learning, AI is paving the way for a new generation of smart nanozymes. This data-centric approach promises major advances in real-world applications.

## 5 Nanozyme combined with other therapies

The integration of metallic hybrid nanozymes with exogenous physical therapies has emerged as a promising strategy for enhanced inflammatory disease treatment. This combined approach leverages the unique catalytic properties of nanozymes alongside the targeted energy delivery of physical modalities, creating a synergistic system that improves both therapeutic efficacy and biological targeting. By simultaneously addressing multiple pathological processes and enhancing localized treatment effects, this paradigm represents a significant advancement beyond conventional monotherapies.

In recent years, numerous studies have demonstrated the successful application of this strategy through various combinations. Metallic hybrid nanozymes have been extensively investigated in conjunction with photothermal therapy (PTT), SDT, PDT,and electrical stimulation (ES) therapy. Furthermore, the integration of nanozymes with multimodal physical treatment approaches has shown particular promise in managing inflammatory conditions, opening new avenues for developing more precise and effective anti-inflammatory platforms (Table 3).

**Table 3:** Metallic hybrid nanozyme-based combination therapy.

Modalities of treatment	Name	Sensitizer	Condition	Bibliography
PTT	UCNPs- ZnO	UCNP	808 nm,3.0 W cm^−2^,30min	[253]
	PISA-Fe	Fe	808 nm,1.0 W cm^−2^,10 min	[254]
	SNPB@Ag	PB	808 nm NIR,1 W cm^−2^,10 min	[255]
	CuS NDs	CuS	808 nm NIR,2.5 W cm^−2^,10 min	[256]
SDT	Pd@Pt-T790	T790	1.0 MHz, 50 % duty cycle, 0.97 W/cm^2^, 8 min	[257]
	TPPS@Au	TPPS	2.85 MHz, 10 Vp-p, 5 min	[258]
	HMMP	PpIX	0.04 MHz, 1 min per cycle, 1.5 W/cm^2^, 5 min	[259]
	MAPC	Ce6	1.0 MHz, 1.0 W/cm^2^, 10 min	[259]
	Au/TNT@PG	TiO_2_	1.0 MHz, 50 % duty cycle, 1.5 W/cm^2^, 8 min	[260]
	g-ZnN_4_-MoS_2_	MoS_2_	1.0 MHz, 50 % duty cycle, 1.5 W/cm^2^, 20 min	[261]
CDT	Mo/GOx@HA	Mo	/	[262]
	RuCu NS	RuCu	/	[160]
ES	GelMA@Ti_3_C_2_/V-OS	MXene、Ti_3_C_2_	100 Hz, 200 mv and square wave,1h	[263]
	rBC/MXene (Ti_3_C_2_T*x*)	Ti_3_C_2_T*x*	200 mV mm^−1^,30 min/day	[264]
Multimodal combination therapy	UCNP@mSiO_2_(RB)-Ag(PTT/SDT)	Ag NP	2.0 W/cm^2^, 10 min	[265]
	CuS/Cur (PTT/PDT/SDT)	Curcumin	1.0 MHz, 50 % duty cycle, 1.0 W/cm^2^, 15 min	[266]
	CuO_2_/TiO_2_ (SDT/CDT)	TiO_2_	1.0 MHz, 1.0 W/cm^2^, 5 min	[163]
	Hb-CFNPs (CDT/PTT)	CuFe_2_O_4_	808 nm,2W/cm^2^,10miin	[267]

### 5.1 Photothermal therapy

PTT is a non-invasive therapeutic strategy that relies on photothermal agents to efficiently convert near-infrared (NIR) light into localized heat, enabling the killing of pathogenic microorganisms and suppression of inflammation [268]. Based on different absorption bands, photothermal agents can respond to multiple NIR windows, including NIR-I (700–950 nm), NIR-II (1000–1350 nm), NIR-III (1600–1870 nm), and NIR-IV (2100–2300 nm). Among these, NIR-I is most commonly used due to the availability of mature laser systems and moderate tissue penetration. However, NIR-II offers lower tissue scattering and allows for higher irradiation doses, thereby exhibiting superior performance [269].

Compared to conventional antibacterial methods, PTT offers unique benefits, including precise site-targeting, low risk of inducing drug resistance, and tunable thermal effects. The local temperature is typically maintained between 45 and 55 °C—sufficient to disrupt microbial membranes while simultaneously enhancing the enzymatic-like catalytic activity of nanozymes (e.g., peroxidase, catalase, superoxide dismutase), thereby boosting the production or scavenging of ROS and strengthening antimicrobial and anti-inflammatory effects [270]. For example, Yang et al. developed a Ti_3_C_2_T_x_-Pt-PEG nanocomposite that exhibited remarkable photothermal conversion efficiency (η ≈ 31.8 %) under NIR laser irradiation. The catalytic activity of the Pt nanozyme was significantly elevated at higher temperatures, promoting efficient •OH generation from H_2_O_2_ for synergistic antibacterial action [271]. Similar design principles have been applied to other photothermal-enhanced antibacterial platforms—such as CeO_2_ and GOx-modified MXene composites—highlighting the expanding promise of nanozyme-assisted PTT [272].

### 5.2 Photodynamic therapy

PDT relies on photosensitizers that are activated under specific wavelengths of light to generate ROS. Photosensitizers can be administered intravenously, sensitizing bacteria to light and converting O_2_ into ROS, thereby causing irreversible damage and apoptosis. However, PDT is less effective in hypoxic environments; therefore, introducing oxygen-producing nanozymes can effectively overcome this oxygen dependency. Hou et al. [273] combined an aggregation-induced emission. photosensitizer with a copper-based nanozyme. The nanozyme generated oxygen at sites of bacterial infection, thereby overcoming the hypoxic limitation of PDT, enhancing antibacterial performance, and alleviating local inflammation in the wound microenvironment. In cellular experiments, treatment with DHTPY-Cu@ZOL resulted in approximately 85.6 % reduction in biofilm formation, highlighting its strong antibiofilm capabilities. In addition to oxygen generation via enzyme-mimicking activity, certain metals can also act as metal-based photosensitizers, enhancing ROS production under UV irradiation to further improve antibacterial effects. For instance, Chen et al. combined copper-cysteine (Cu-Cy) nanosheets with Ag NPs to form a Cu-Cy-PEG@AgNPs composite, which efficiently generated ROS under UV light. At a low concentration of 25 μg/mL, it achieved nearly 100 % bactericidal efficiency, demonstrating excellent antibacterial activity against both Gram-negative and Gram-positive bacteria [274].

### 5.3 Sonodynamic therapy

Inspired by PDT, SDT utilizes low-frequency ultrasound to activate sonosensitizers for ROS generation. SDT offers the advantage of non-radiative deep tissue and organ penetration. Compared to PDT, SDT allows deeper therapeutic action under conditions of lower tissue attenuation. Although the exact mechanism of SDT remains unclear, it is widely believed that ROS production primarily stems from three mechanisms: cavitation effects, localized thermal effects, and mechanical damage. Among these, cavitation is considered the core mechanism. When sonosensitizers are activated by ultrasound, they transition from the ground state to an excited state, which can transfer energy to surrounding molecular oxygen, generating ROS via sonoluminescence or thermal decomposition. This process involves the formation, growth, and collapse of microbubbles, as well as their sustained oscillation.

Sonosensitizers play a key role in antibacterial and anti-inflammatory therapy. To date, a variety of sonosensitizers have been employed for SDT. In recent years, their integration into nanomaterials has increased to improve therapeutic outcomes. In addition to organic sonosensitizers, certain metal ions have also been identified as effective sonosensitizers. Inorganic agents such as Ti-based nanoparticles possess distinctive band structures and relatively high chemical/physiological stability. However, traditional TiO_2_ nanoparticles show limited SDT performance due to the rapid recombination of electrons (e^−^) and holes (h^+^) in their band structure. Several strategies have been proposed to enhance ROS generation by promoting effective e^−^/h^+^ separation, such as creating oxygen vacancy layers, combining TiO_2_ with noble metals (e.g., Pt, Au, Ag), or doping TiO_2_ with transition metals like Fe and V. In addition to enhanced SDT performance, metal-based nanozymes can also amplify ROS generation and antimicrobial activity. Sun et al. [275] synthesized Si-Pt nanocomposites (Si-Pt NCs) by in situ reduction of Pt nanoparticles grown on silicon nanowires (SiNWs). Thanks to the excellent catalytic properties of Pt NPs and the mesoporous architecture of SiNWs, the resulting Si-Pt NCs exhibited significantly improved SDT and Chemodynamic therapy (CDT) performance compared to pure Pt NPs. Additionally, Chen et al. [276] constructed a composite material (Au/BP@MS) by integrating Au NPs, manganese dioxide (MnO_2_), and biodegradable ultrathin black phosphorus (BP) nanosheets. The incorporation of Au NPs with BP nanosheets reduced BP's bandgap, enhancing e^−^/h^+^ separation efficiency and thus amplifying its sonodynamic activity.

### 5.4 Chemodynamic therapy

CDT eradicates bacteria by generating •OH through Fenton or Fenton-like reactions. With the rapid advancement of nanomaterials, CDT has attracted wide research interest due to its unique advantages, including high selectivity, minimal side effects, and the absence of a need for external activation. The core of the Fenton reaction is the catalytic conversion of H_2_O_2_ by Fe^2+^ to produce hydroxyl radicals. Recent studies have shown that other metal ions such as Cu^2+^, Mn^2+^, Co^2+^, and Ti^3+^ can also catalyze H_2_O_2_ to generate •OH via similar Fenton-like mechanisms. Liu et al. [277] developed a nanozyme by modifying hollow polydopamine (HPDA) with Cu-Fe bimetallic peroxides (CFp), termed CFp/HPDA. In the acidic bacterial infection microenvironment, CFp can release Cu^+^ and Fe^2+^ ions, triggering Fenton-like catalytic reactions and producing high levels of •OH for effective treatment of infected wounds. Despite its promise, CDT alone primarily relies on ROS-mediated mechanisms to kill pathogens and modulate inflammation. However, due to the complex and heterogeneous nature of microbial infections and inflammatory processes, monotherapy may fall short in addressing multifaceted clinical scenarios [278]. As a result, current research increasingly focuses on integrating CDT with other therapeutic modalities to harness synergistic effects and achieve more robust and comprehensive treatment outcomes.

### 5.5 Electrical stimulation therapy

In recent years, electrical stimulation (ES) therapy has gradually been applied to fields such as chronic inflammation and wound healing. The application of an external electric field can not only enhance the catalytic activity of nanozymes but also promote the release of active molecules at targeted sites. Meanwhile, the accompanying electrothermal effect can improve the local microenvironment, facilitating cell migration, angiogenesis, and antioxidant responses, thereby significantly enhancing tissue regeneration [279]. Hwang et al. [280] developed an Electrically Responsive Multifunctional Patch (ERMP) that integrates self-powered ES, ROS regulation, and bioactive metal ion release. This system utilizes a triboelectric nanogenerator (TENG) to harvest biomechanical energy from body movement, generating self-powered ES that is delivered to the wound site via miniature magnesium (Mg) microneedles. In parallel, the applied electric field induces ion electroosmosis, facilitating the efficient release of Mg^2+^ to further modulate the local wound environment. Notably, the incorporation of Prussian Blue (PB) into the system not only improved the electrical output of the TENG, but also enabled precise ROS regulation via carbon-nitrogen vacancies on its modified surface. In vivo experiments demonstrated that this multifunctional patch markedly accelerated wound healing, with closure rates nearly 9 times faster than those observed in control groups. This enhancement was attributed to the synergistic effects of ES, ROS modulation, and controlled Mg^2+^ release—highlighting its potential to overcome the inherent limitations of traditional therapies.

### 5.6 Multimodal combination therapy

Due to the complexity of the therapeutic microenvironment, monotherapies often face limitations in clinical applications. As a result, researchers are increasingly integrating multiple treatment modalities to achieve enhanced therapeutic outcomes. Currently, multimodal platforms that combine PTT, SDT, PDT, and nanozyme-based catalytic therapy are rapidly evolving.

PTT utilizes photothermal agents to convert light into heat, inducing thermal ablation to eliminate bacteria. Additionally, elevated local temperatures can promote Fenton or Fenton-like reactions. Thus, PTT is often combined with PDT and CDT to achieve synergistic effects. The generation of ROS is the primary mechanism behind nano-dynamic therapies. PDT and CDT generate ROS via photosensitizers and Fenton/Fenton-like reactions, respectively. However, in CDT, •OH radicals are often depleted by excess GSH, reducing efficacy. Therefore, combining CDT and PDT with nanozymes to overcome these shortcomings and enhance treatment efficiency has become a key research focus. These integrated systems not only enable layered treatment of superficial and deep inflammatory lesions but also offer functionalities like imaging-guided diagnosis and controlled drug release, paving the way for precise, efficient, and tunable anti-inflammatory therapies.

For instance, Song et al. [281] developed a NIR-responsive hybrid nanozyme system, Zn SACs@CuO_2_, for synergistic photothermal and catalytic therapy. This system was fabricated using ZIF-8 as a template to create Zn single-atom catalysts (SACs), followed by in situ CuO_2_ shell formation under Cu^2+^ and H_2_O_2_ conditions, yielding uniform dodecahedral nanostructures. TMB oxidation results showed that Zn SACs@CuO_2_ had 1.15 × higher catalytic activity than Zn SACs and 1.39 × that of Zn NPs, confirming enhanced peroxidase-like activity and photothermal stability, improving overall PTT/CDT therapeutic outcomes. Zhai et al. [282] constructed bimetallic single-atom nanozymes (FeCu BSNs) with Fe and Cu active centers for effective infection treatment. The hybridization of Fe and Cu resulted in strong POD-like activity (752.25 U mg^−1^), over twice that of pure Fe nanozymes (323.45 U mg^−1^). Incorporating Cu into the Fe site improved light absorption and spin-orbit coupling, boosting PTT performance. The system demonstrated a photothermal conversion efficiency of 56.26 %, nearly double that of Fe-only (29.69 %) and Cu-only (25.5 %) counterparts, and also significantly enhanced POD activity under laser irradiation, achieving high antibacterial and wound healing efficacy at low doses.

Beyond basic CDT-PTT combinations, Hu et al. [283] developed a multifunctional nanozyme (PB@Cu^2+^/ZnP NPs) that integrates chemotherapy, CDT, and CTT within a single nanoplatform, exhibiting excellent biological activity. Other researchers have explored more holistic nanozyme designs. For instance, Mei et al. [284] proposed a BME-responsive copper-doped polyoxometalate (Cu-POM) system that synergizes mild PTT with macrophage immune modulation to eliminate biofilm-associated infections (BAIs) at multiple stages. Wang et al. [285] developed a multifunctional wound dressing (PSAU@CuPDA) using a hydrogel matrix of SBMA and AM, crosslinked with HDPU and doped with CuPDA NPs. The CuPDA and HDPU enhanced the hydrogel's adhesion, photothermal, and electrical properties. Combined PTT/PDT/CDT/EST therapy using this dressing achieved ∼93.15 % wound healing efficiency, while adding ES further increased healing to ≈98.96 %, making it a promising all-in-one platform for antimicrobial wound treatment.

## 6 Application of metallic hybrid nanozyme in inflammatory diseases

### 6.1 Inflammatory bowel disease

IBD is a non-specific, chronic inflammatory condition of the gastrointestinal tract, closely associated with oxidative stress, dysbiosis of the gut microbiota, and disruption of the pro-inflammatory microenvironment in the intestine [286,287]. During IBD flare-ups, large numbers of inflammatory cells and neutrophils infiltrate the intestinal mucosa and accumulate at inflamed sites, releasing abundant ROS and cytokines. This leads to epithelial cell death and compromises the integrity of the intestinal barrier, thereby promoting pathogenic invasion. Metallic hybrid nanozymes have shown great potential in IBD therapy by scavenging ROS and promoting mucosal repair.

**Fig. 6:**
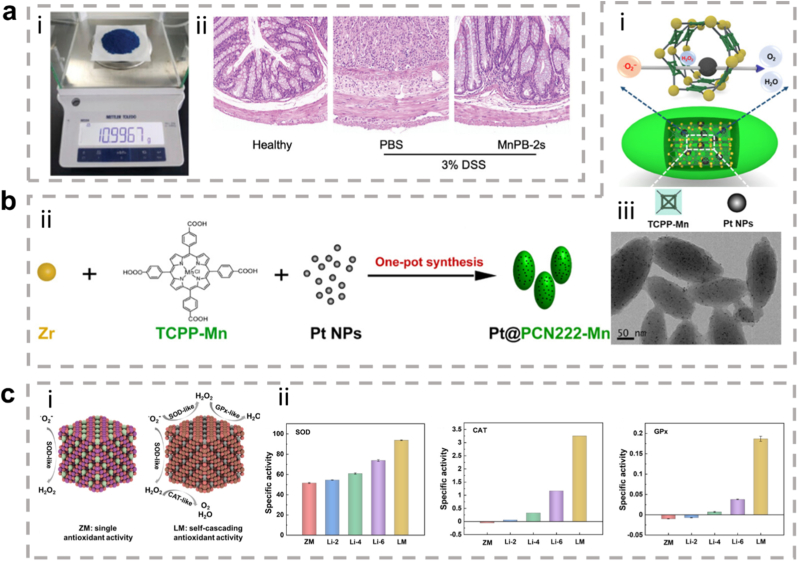
(a) Manganese iron Prussian blue nanozymes (i) Photographs of massively prepared MnPB-2 nanoases (ii) H&E staining of colon sections [289].Copyright 2025 Ivyspring International Publisher. (b) Integrated nanozymes with cascade anti-ROS activity for IBD treatment (i) Schematic diagram of the design of PCN222-Mn MOFs (ii) Synthesis process of Pt@PCN222-Mn (iii) TEM images of Pt@PCN222-Mn-5 [22].Copyright2025 American Association for the Advancement of Science. (c) Schematic diagram of valence engineering design of manganese oxide spinel for enhanced IBD treatment from cascaded antioxidant nanozymes (i) Comparison of antioxidant activity before and after valence engineering (ii) Comparison of antioxidant activity of ZM, Li-2, Li-4, Li-6 and LM. [219].Copyright 2022 Wiley-VCH GmbH. (For interpretation of the references to color in this figure legend, the reader is referred to the Web version of this article.)

Among various metallic nanozymes, PB has been extensively studied due to its facile synthesis, stable performance, and FDA approval, and it shows promising application potential in IBD treatment. However, in complex models such as UC and Crohn's disease (CD), the single structure and function of PB nanozymes are no longer sufficient to cope with the intricate pathological environment. Multi-metal synergistic catalysis is an effective strategy for achieving cascade reactions. Based on this, Zhao et al. [288] employed a hydrothermal method using Mn^2+^, [Fe(CN)_6_]^4−^, and polyvinylpyrrolidone (PVP) to construct a manganese-Prussian Blue nanozyme (MPBZ) with multi-enzyme activities. The low redox potentials and variable valence states of Mn(II) and Fe(II) imparted MPBZ with both CAT- and SOD-like activities and enhanced its redox buffering capacity, resulting in excellent anti-inflammatory performance. Despite its advantages, PB nanozymes still face technical challenges in large-scale production. To address this, Hu et al. [289] developed a one-pot synthesis strategy in which MnSO_4_ and K_4_Fe(CN)_6_ were mixed in citric acid to produce a porous MnFe-PB (MnPB) nanozyme. This method achieved an unprecedented high yield of approximately 11 g per batch, representing a 100-fold increase compared to small-scale synthesis. Importantly, the large-batch product showed minimal changes in structure and elemental composition and exhibited comparable antioxidant capacity and therapeutic performance to the small-scale product (Fig. 6a). Animal experiments demonstrated that oral administration of MnPB at 10 mg/kg for three consecutive doses significantly alleviated acute UC symptoms in mice, confirming the feasibility of scaling up PB-like nanozyme production without compromising therapeutic potential and laying the groundwork for future large-animal studies.

Beyond PB-based nanozymes, various manganese-based hybrid nanozymes have also demonstrated excellent efficacy in IBD models. Liu et al. [22]developed an integrated SOD/CAT-mimicking cascade nanozyme (Pt@PCN222-Mn) using a one-pot synthesis method, where catalase-like Pt nanoparticles and SOD-mimicking Mn(III) porphyrins were incorporated into a Zr-based MOF, PCN222. This structure exhibited enhanced SOD and CAT-like activities. The pore confinement effect enabled high-density Pt loading and prevented aggregation caused by small particle size, thereby enhancing its catalytic synergy (Fig. 6b). In vivo, Pt@PCN222-Mn-5 effectively treated UC in mice, as evidenced by reduced weight loss, longer colon length, lower levels of TNF-α and IL-1β, and improved histological appearance compared with other groups. To further explore the relationship between manganese valence states and antioxidant performance, Wang et al. [219] used ZnMn_2_O_4_ as a model and applied a valence engineering strategy to investigate the effect of Mn valence at octahedral sites. They found a positive correlation between antioxidant activity and Mn4+ content and developed a self-cascading antioxidant nanozyme, LiMn_2_O_4_. By gradually doping with Li, the average Mn valence increased from +3 to +4, transforming ZnMn_2_O_4_—which had only SOD-like activity—into LiMn_2_O_4_, which possessed SOD-, CAT-, and GPx-like activities. The resulting nanozyme exhibited strong antioxidant and therapeutic effects both in cell-level assays and in IBD models, along with a pronounced dose advantage in animal studies (Fig. 6c).

These studies collectively demonstrate that the introduction of different metal dopants can not only impart new enzymatic activities but also improve material properties through mechanisms such as surface charge modulation. Beyond manganese-based systems, doping with other metals can also optimize nanozyme performance. For example, Li et al. [290] synthesized Au/CeO_2_ core-shell porous nanozymes, where the structure provided a high surface-area-to-volume ratio for catalytic site exposure and facilitated Ce(III)/Ce(IV) redox cycling, thereby enhancing CAT- and SOD-like antioxidant activity. After coating with negatively charged hyaluronic acid (HA), Au/CeO_2_@HA accumulated on the positively charged inflamed mucosa following oral administration, resulting in targeted action and effective mitigation of colon damage in mice with acute colitis. Similarly, Cui et al. [99] reported a highly biocompatible iodine-copper-zinc co-doped carbon dot nanozyme (Cu,Zn,I-CDs). Iodine doping helped neutralize the positive charge generated by Cu and Zn under acidic conditions, allowing Cu, Zn, I-CDs to exhibit enhanced SOD-, CAT-, and GPx-like activity. These nanozymes also suppressed pro-inflammatory cytokines (IL-1β, IL-6, TNF-α) and upregulated antioxidant gene expression, demonstrating comprehensive anti-inflammatory effects. Notably, Cu, Zn, I-CDs exhibited strong environmental tolerance and retained higher enzyme-mimicking activity than natural SOD under extreme acidic or alkaline conditions. Zhao et al. [291] constructed a pH-responsive nanozyme (NiCo_2_O_4_@PVP) using a stepwise strategy. First, a layered double hydroxide (LDH) of NiCo was synthesized using ZIF-67 as a template via ion exchange, followed by calcination to produce NiCo_2_O_4_ nanoparticles. Finally, PVP was used to coat the NiCo_2_O_4_ nanoparticles, enhancing their biocompatibility and acid resistance. The resulting nanozyme contained oxygen vacancies that facilitated oxygen compound capture, while Co^3+^/Co^2+^ and Ni^3+^/Ni^2+^ redox pairs provided abundant catalytic sites. The NiCo_2_O_4_@PVP nanozyme exhibited SOD-, CAT-, POD-, and OXD-like activities and demonstrated significant pH-dependent performance, indicating broad application value and great potential for development.

In summary, strategies such as multi-metal synergy and valence-state modulation can not only expand the enzymatic repertoire of nanozymes but also improve their biodistribution and stability through structural engineering and surface modification. These approaches provide a multidimensional design rationale to advance the clinical translation of metallic hybrid nanozymes for IBD treatment.

### 6.2 Neurological disorders

Neurological diseases, particularly those significantly influenced by inflammation—such as AD, traumatic brain injury, and stroke—are closely related to elevated levels of local ROS, neuronal damage, and chronic inflammation. Due to the high sensitivity of neural tissue to oxidative stress, and the often latent and slow-progressing nature of many neuroinflammatory conditions, traditional treatment methods frequently lack targeting ability and real-time monitoring capacity. Nanozymes not only exert therapeutic effects through their antioxidant activity but can also achieve dynamic regulation of inflammatory responses and precise intervention by monitoring pathological changes in real time and selectively eliminating inflammation-related disease markers. Therefore, nanozymes with theranostic potential offer a new strategy for the precise treatment and effective monitoring of central nervous system disorders.

#### 6.2.1 Alzheimer's disease

AD is an irreversible, progressive neurodegenerative disorder of the central nervous system, clinically characterized by memory loss, cognitive impairment, and behavioral decline [292,293]. Pathologically, AD is marked by extracellular accumulation of β-amyloid (Aβ) plaques, intracellular neurofibrillary tangles caused by hyperphosphorylation of tau protein, and progressive neuronal loss [294]. Dysregulation of dopamine (DA) is considered one of the major contributors to AD, and both detection and clearance of its related biomarkers play essential roles throughout the treatment process. Additionally, oxidative stress is a critical factor in AD pathogenesis. Accumulated ROS can induce redox imbalance and neurotoxicity mediated by Aβ and tau proteins, further exacerbating neuronal apoptosis. Aβ can also bind to metal ions such as Cu^2+^ and Fe^3+^, thereby promoting ROS production and accelerating disease progression [295].

**Fig. 7:**
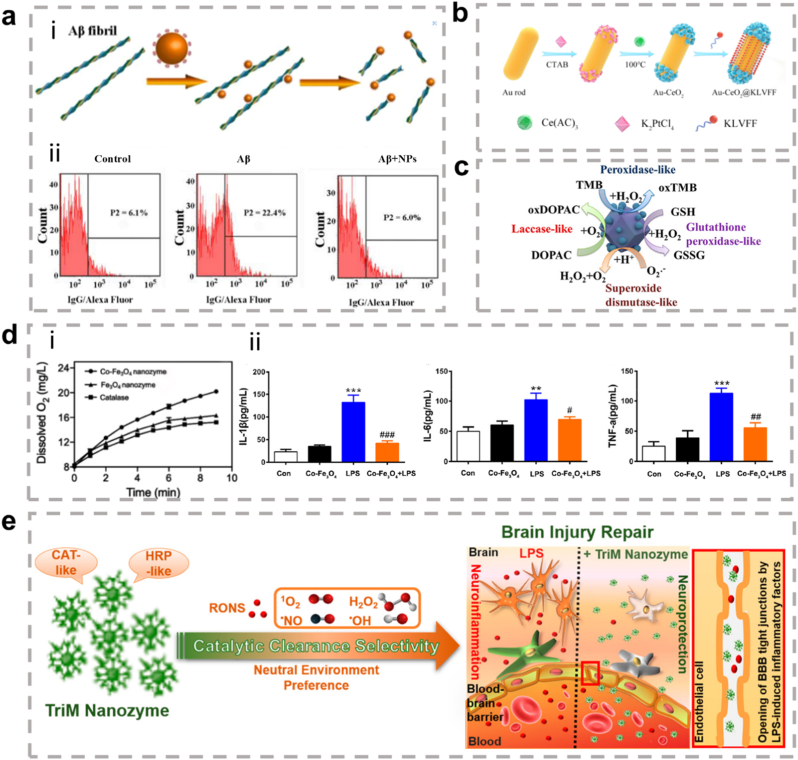
(a) Ceria/POMs hybrid nanoparticles as artificial metalloproteases for treating Aβ-induced neurotoxicity (i) Schematic illustration of the synthesis of CeO_2_NP@POMs.(ii). Flow cytometry analysis of CD11b-positive cell populations [294]. Copyright 2016 Elsevier Ltd. All rights reserved. (b) Schematic synthesis route of K-CAC nanocomposites [296]. Copyright © 2022 American Chemical Society. (c) Design schematic of the ZIF-67/Cu_0_._76_Co_0_._24_O_4_ nanozyme [298]. Copyright 2020 American Chemical Society. (d) Co-doped Fe_3_O_4_ nanozyme alleviates ischemic stroke.(i) Dissolved oxygen production under different treatment groups. (ii) Inhibition of H_2_O_2_-induced inflammatory cytokine expression by Co-Fe_3_O_4_ nanozyme [299]. Copyright 2021 The Authors.(e) Schematic mechanism of brain injury repair by triM nanozyme with high catalytic activity and neutral environment preferenc [300]. Copyright2019 American Chemical Society.

Effectively degrading Aβ and interrupting its toxic cascade have become key challenges in tackling the complex pathology of AD. To this end, Guan et al. [294] designed a cerium dioxide/polyoxometalate hybrid nanozyme (CeO_2_NP@POMs) with a Wells-Dawson structure that exhibits both protease-like and SOD-like activities. Benefiting from the large molecular size, high negative charge, and multiple coordination sites of the Wells-Dawson polyoxometalate anion, CeO_2_NP@POMs demonstrated high proteolytic activity toward Aβ, enabling it to cross the blood-brain barrier (BBB), degrade Aβ aggregates, and reduce intracellular ROS. In cellular studies, CeO_2_NP@POMs significantly suppressed Aβ-induced microglial activation, promoted neuronal proliferation, and downregulated inflammatory responses, exhibiting promising therapeutic potential (Fig. 7a). Ge et al. [296] reported a nanocomposite, KLVFF@Au-CeO_2_K-CAC, synthesized by depositing CeO_2_ at the ends of gold nanorods and modifying the rod center with the Aβ-recognition peptide KLVFF. This system enabled efficient targeting and clearance of Aβ aggregates both in vitro and in vivo (Fig. 7b). In addition, the structure enhanced local photothermal effects, which in turn activated the SOD- and CAT-like enzymatic activities of CeO_2_ under near-infrared (NIR) irradiation, significantly boosting ROS-scavenging capacity. The spatial separation of functional domains further improved catalytic activity, photothermal conversion efficiency, and BBB permeability. Sher et al. [297] developed Ag/Au nanozymes via green synthesis that exhibited significant inhibitory effects in acetylcholinesterase activity assays, suggesting their potential as screening tools or adjunctive treatments for AD.

Beyond direct therapeutic intervention, the use of nanomaterials to detect neurotransmitters and disease biomarkers is a critical strategy for early diagnosis of AD. Zhu et al. [301] developed a Prussian Blue-MoS_2_ composite nanozyme platform for highly sensitive dopamine (DA) detection. Subsequently, Liu et al. [298] designed a “raisin pudding”-like nanozyme, ZIF-67/Cu_0_._76_Co_0_._24_O_4_, which exhibited four enzyme-mimicking activities (SOD, CAT, GPx, and laccase-like), and enabled real-time monitoring of DOPAC, a metabolic product associated with AD, in the living brain (Fig. 7c). In this system, ZIF-67 served as a cobalt source, while Cu doping imparted laccase-like activity essential for DOPAC oxidation, supporting in vivo neurotransmitter detection. Liu et al. [302] further constructed an AuBP@Pt nanozyme platform for early diagnosis of AD, capable of precise detection of the high-risk genetic marker apolipoprotein E4 (APOE4), offering a new strategy for disease prediction and stratification. For Aβ monitoring, Zhou et al. [303] developed porous bimetallic ZnO-Co_3_O_4_ nanocages with high peroxidase-like activity. The catalytic activity of ZnO-Co_3_O_4_NCs was significantly inhibited by Aβ monomers, enabling their use in colorimetric assays for Aβ detection.

#### 6.2.2 Stroke

Stroke is an acute cerebrovascular event with high rates of disability and mortality, primarily classified into ischemic and hemorrhagic types [304]. Among them, ischemic stroke accounts for over 85 % of cases and is typically caused by vascular occlusion, leading to interruption of blood supply to brain tissue, which can result in severe disability or death [305]. Clinically, timely intravenous thrombolysis or mechanical thrombectomy is used to achieve reperfusion and restore blood flow to the ischemic area. However, reperfusion introduces oxygen and glucose back into the ischemic brain tissue, triggering the excessive production of ROS and reactive oxygen and nitrogen species (RONS) [306]. These ROS can stimulate the expression of adhesion molecules and cytokines, directly or indirectly damaging tissues and cells, resulting in reperfusion injury. Therefore, timely regulation of ROS generation during ischemia-reperfusion injury is a key factor in the effective treatment of ischemic stroke.

However, due to the protective function of the BBB, many nanozymes that exhibit neuroprotective effects in vitro are unable to reach the target site in vivo, and only a few demonstrate neuroprotective efficacy in living systems. Liu et al. [299] developed a Co-doped Fe_3_O_4_ nanozyme that exhibits both POD- and CAT-like activities. Under neutral conditions, this nanozyme showed a 100-fold higher affinity for H_2_O_2_ compared with pure Fe_3_O_4_, and was capable of suppressing H_2_O_2_-induced neurotoxicity, neuroinflammation, and lipopolysaccharide production in HT22 cells (Fig. 7d). The therapeutic mechanism may involve the uptake of Co-Fe_3_O_4_ by neurons, astrocytes, microglia, and endothelial cells at the ischemic boundary of the brain, thereby exerting its neuroprotective effects. Notably, this nanozyme is capable of crossing the BBB and accumulating in brain tissue after ischemic injury, while under physiological conditions it does not cross the BBB and instead accumulates in the liver. In addition to heterometallic compositions, atomically dispersed nanozymes composed of multiple atoms of the same metal have also shown promising performance. Such single-atom nanozymes (SAzymes) offer uniform active centers and high atomic utilization efficiency, which can be further enhanced to achieve strong synergistic effects. Compared with other metallic compositions, triM nanozymes demonstrate superior electron capture ability and greater affinity for ROS and RONS under neutral conditions. For instance, Mu et al. [300] developed the Pt-Pd-Mo nanocomposite, exhibiting strong preference for neutral environments and significantly improved therapeutic outcomes in brain injury models (Fig. 7e).

It is worth noting that the application of nanozymes in ischemia-reperfusion injury is not limited to the brain. Liu et al. [307] designed a manganese-chelated coordination nanoplatform (MCN) capable of targeting infarcted myocardial tissue with high specificity. This system effectively scavenged ROS produced during myocardial infarction and reperfusion, alleviated oxidative damage, and showed excellent cardiac repair effects. Similar strategies have been extended to intestinal and hepatic ischemia-reperfusion models. For example, Eu-CeO_2_ nanozymes protected intestinal barriers in a gut ischemia model by mitigating oxidative stress and inflammation [308]. MnOx-CeO_2_ nanozymes also demonstrated strong targeting ability and significantly alleviated liver reperfusion injury [309].

### 6.3 Ocular inflammatory diseases

Ocular diseases such as Dry Eye Disease (DED), Diabetic Retinopathy (DR), and Bacterial Keratitis (BK) are typically multifactorial, often accompanied by oxidative stress, inflammatory responses, and mitochondrial dysfunction. In recent years, nanozymes have shown great potential in the treatment of ocular diseases due to their enzyme-mimicking catalytic properties, ease of preparation, catalytic stability, and high resistance [310].

**Fig. 8:**
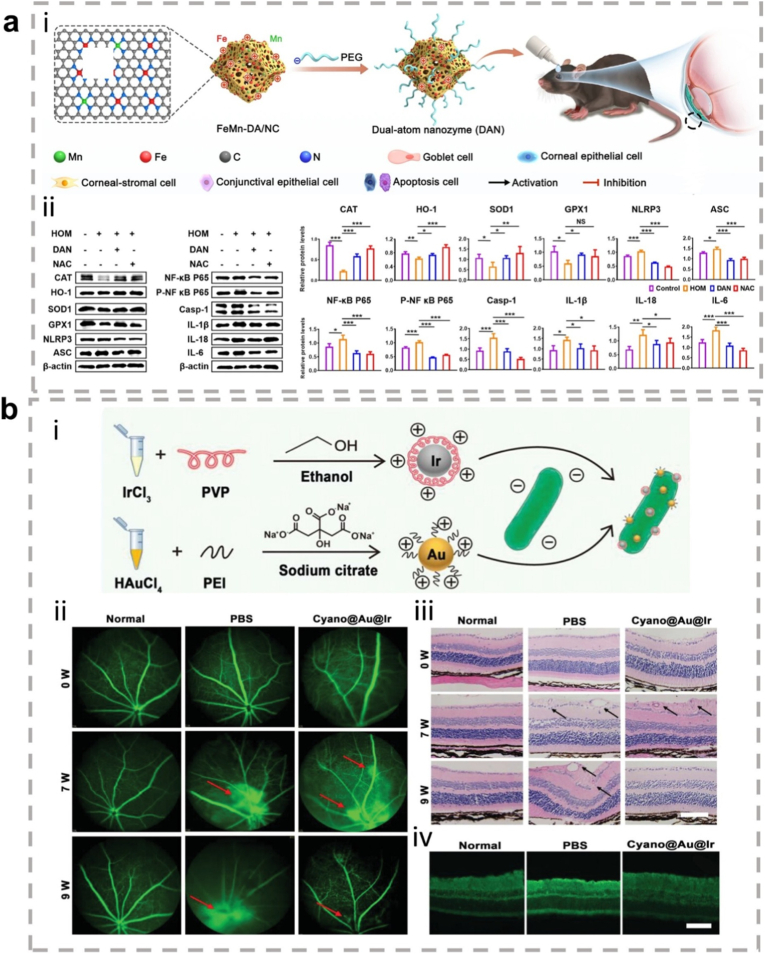
(a) (i) Schematic illustration of the diatomic nanozyme. (ii) The protein expression levels of SOD1, CAT, GPX1, HO-1, NLRP3, ASC, Caspase-1, IL-1β, IL-18, IL-6, NF-κB P65, and P-NF-κB P65 in cell lysates derived from HCE-2 cells were analyzed by western blotting and densitometry analyses of the western blotting results. Data are presented as mean ± SD [165]. (b) (i) Schematic illustration of the synthesis process of Cyano@Au@Ir.(ii) Fundus fluorescein angiography of retinal cross-sections at weeks 0, 7, and 9 after citrate buffer/STZ injection. (iii) H&E staining of retinas following subretinal injection of PBS or Cyano@Au@Ir. (iv) Retinas labeled with pimonidazole under different treatments at 2 weeks post-treatment.

DED is a multifactorial disorder of the ocular surface, mainly caused by tear film instability and subsequent hyperosmolarity. This activates cellular stress pathways, leading to the secretion of various pro-inflammatory mediators that recruit additional inflammatory cells to the ocular surface, thereby exacerbating the inflammatory response and forming a self-sustaining vicious cycle. To break this cycle, Zhu et al. embedded Fe and Mn dual metal single atoms into nitrogen-doped carbon materials and modified them with hydrophilic polymers, successfully preparing a DAN, FeMn-DAN [165]. This DAN mimicked the activity of five enzymes—POD, CAT, OXD, SOD, and GPx—exhibiting excellent antioxidant activity. FeMn-DAN significantly cleared ROS induced by hyperosmolarity, inhibited the activation of NLRP3 inflammasomes, reduced the expression of pro-inflammatory factors such as IL-1β, IL-18, IL-6, and NF-κB P65, and alleviated inflammation in HCE-2 cells. Moreover, FeMn-DAN also restored the expression of antioxidant proteins, including CAT, HO-1, GPX1, and SOD1, enhancing the cells' antioxidant capacity (Fig. 8a).

DR is primarily characterized by hyperglycemia and hypoxia in the microvascular regions due to impaired glucose metabolism, making it a leading cause of blindness. In the early stages, microaneurysms appear, and in later stages, hard exudates, soft exudates, neovascularization, and vitreous hemorrhage may occur. Reducing glucose levels and oxidative stress in the microenvironment is considered an effective strategy for controlling the progression of DR. Wang et al. developed a novel photosynthetic-biohybrid system that regulates the DR microenvironment by continuous oxygen supply and nanozyme cascaded reactions [311]. This system used cyanobacteria as a carrier, and in situ reduction reactions loaded Au NPs with glucose oxidase-like activity and iridium nanoparticles (Ir NPs) with peroxidase-like activity (Cyano@Au@Ir). Au NPs first degraded glucose into hydrogen peroxide, which was subsequently decomposed by Ir NPs into water and oxygen, completing the cascaded glucose-lowering reaction. Additionally, Cyano, as the carrier, continuously generated oxygen under visible light, improving the hypoxic environment in the DR region. The experiment showed that DR-related neovascular leakage was completely suppressed and effectively reversed, with the vascular morphology nearly restored (Fig. 8b). This study demonstrated the potential of metal-hybrid nanozymes in synergistic treatment when combined with microorganisms.

BK is caused by bacterial infections in the eye, and in severe cases, it can lead to vision impairment or blindness. Current treatments primarily rely on local drug delivery or intrastromal antibiotic injections. However, due to the anatomical structure of the cornea and dilution by the tear film, local drug bioavailability is low, and antibiotic abuse has led to the emergence of resistant bacteria, posing challenges to traditional treatments. Although nanozymes show potential in corneal wound healing, existing nanozymes lack sufficient catalytic activity and the ability to penetrate bacterial biofilms, limiting their effectiveness in treating BK. To address this issue, Tu et al. loaded Cu single-atom nanozymes (Cu-SAzymes) and aminated dextran (Dex-NH_2_) onto ZnFe layered double hydroxide (ZnFe-LDH) nanosheets to form a nanozyme, DT-ZnFe-LDH@Cu, which exhibited POD, OXD, and CAT-like catalytic activity [312]. The surface-modified Dex-NH_2_ enabled DT-ZnFe-LDH@Cu to penetrate biofilms and adsorb to extracellular polymeric substances (EPS) produced by bacteria. Additionally, DT-ZnFe-LDH@Cu promoted M1 macrophage polarization to M2 macrophages, reduced inflammation, and lowered α-SMA expression, promoting wound healing without scar formation. Combination therapies have also been an effective strategy to improve antibacterial performance. Wang et al. developed a Pd-doped titanium oxide nickel nanozyme (PdOV-TiOX) with oxygen vacancies by defect engineering [313]. The introduction of oxygen vacancies significantly enhanced its peroxidase-like activity, accelerating the generation of •OH and enhancing its photothermal response. Under NIR-II laser excitation, this nanozyme could synergistically exert both PTT and CDT effects, showcasing multiple antibacterial actions. In addition to treating keratitis, this nanozyme also showed promising therapeutic effects in a pneumonia model, indicating potential for controlling cross-organ infections.

### 6.4 Periodontal disease

Periodontal disease is a chronic inflammatory condition induced by the host immune response and has been identified as the sixth most prevalent non-communicable disease globally [314]. Unlike other inflammatory disorders, its pathogenesis is uniquely initiated by bacteria within dental plaque. While the immune response helps control infection, it can also lead to immune suppression and bacterial overgrowth, ultimately causing destruction of periodontal tissues and tooth loss [315]. Therefore, treatment of periodontitis involves the dual challenge of antibacterial and anti-inflammatory interventions. Numerous recent studies have reported promising strategies in this area.

**Fig. 9:**
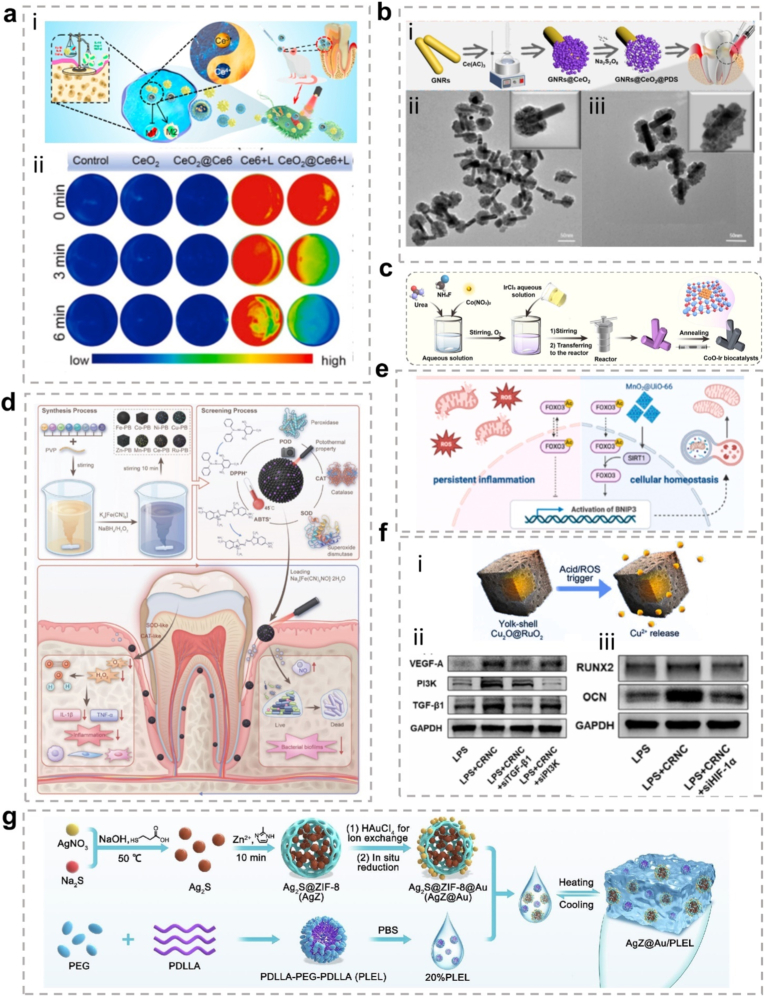
(a) Multifunctional nanocomposites based on nanoceria. (i) Schematic illustration of the design of CeO_2_@Ce6 nanocomposite. (ii) Representative in vitro fluorescence images of ROS generation by different nanoparticles captured using an in vivo imaging system [316]. Copyright 2020 Elsevier Ltd. (b) Design of h-GNRs@CeO_2_@PDS NC. (i) Schematic depiction of h-GNRs@CeO_2_@PDS NC, featuring functionalities for combating biofilm infections and mitigating aPTT, PCAT, and ROS storms. (ii, iii) Representative TEM images of h-GNRs@CeO_2_ and t-GNRs@CeO_2_ [317]. Copyright 2024 Elsevier B.V. (c) Schematic illustration of the synthesis of CoO-Ir [320]. Copyright 2023 AmerSociety. (d) Schematic depiction of the development and evaluation of a Prussian blue-based nanozyme library for periodontitis treatment [215]. Copyright 2024 Wiley-VCH GmbH. (e) Schematic representation showing how MU enhances mitophagy and mitochondrial quality via FOXO3 deacetylation mediated by SIRT1 [321]. Copyright2025 Elsevier Ltd. (f) Intelligent multifunctional Cu_2_O@RuO_2_ nanozyme for promoting angiogenesis and osteogenesis in periodontitis. (i) Schematic illustration of the drug release behavior of CRNC nanozyme. (ii) Immunoblotting results of VEGF-A, PI3K, and TGF-β1 expression after knockdown of TGF-β1 or PI3K in HUVECs. (iii) Immunoblotting of OCN and RUNX2 in PDLSCs after HIF-1α knockdown under specified treatments [322]. Copyright 2025 Elsevier B.V. (g) Schematic illustration of the synthesis of AgZ@Au/PLEL hydrogel [323]. Copyright 2025 Published by Elsevier B.V. (For interpretation of the references to color in this figure legend, the reader is referred to the Web version of this article.)

CeO_2_ and its derivatives have shown extensive application potential in the treatment of periodontitis. Sun et al. [316] coated cerium oxide nanoparticles with a red light-activated photosensitizer, Ce6, to construct CeO_2_@Ce6 hybrid nanozymes with SOD- and CAT-like activity. This design also introduced antimicrobial photodynamic therapy (aPDT) to address excessive local accumulation of ROS. Under red light irradiation, the system enables efficient bacterial killing via aPDT, followed by ROS scavenging and immune modulation through CeO_2_, achieving a “bacteria-killing followed by anti-inflammatory” synergistic therapeutic effect (Fig. 9a). Building on this, Li et al. [317] developed a more structurally complex mushroom-shaped Janus nanostructure (h-GNRs@CeO_2_), in which multilayered porous CeO_2_ was half-wrapped at one end of gold nanorods (GNRs) (Fig. 9b). The structure leveraged the high photothermal conversion efficiency of GNRs and incorporated photocatalytic antibacterial therapy (PCAT) to treat microbial infections effectively. Notably, precise regulation of cerium ion valence states within CeO_2_ further enhanced its POD-like activity and significantly improved antibacterial and antioxidant performance. Morphology is also a key determinant of nanozyme catalytic properties. Variations in CeO_2_ structure influence the redox cycling between Ce^3+^ and Ce^4+^, which directly impacts its anti-inflammatory and antibacterial activities. Li et al. [318] systematically compared three CeO_2_-modified Ti-based materials—nanorods, nanocubes, and nanooctahedrons—and found the octahedral structure most effective in suppressing periodontal inflammation and biofilm formation. Extending this structural advantage, Cai et al. [319] prepared Pd@Ir nanooctahedrons, which exhibited higher oxidase activity and bactericidal efficacy than their cubic counterparts, confirming the generality of structure-dependent catalytic performance. Furthermore, Xie et al. [320] developed CoO-Ir nanozymes in which Ir nanoclusters were uniformly supported on the CoO lattice due to strong electronic coupling and charge transfer. These clusters demonstrated cascade SOD- and CAT-like activity (Fig. 9c), excelling in ROS clearance, inflammation regulation, and osteogenic differentiation, thus showing significant promise in periodontitis treatment.

Beyond ROS, nitric oxide (NO) also plays a dual role in periodontal tissue: low concentrations promote vasodilation, glycolysis-driven osteogenesis, and soft tissue repair, while high concentrations induce cytotoxicity and amplify inflammation. Exogenous NO delivery is considered a potent strategy for periodontitis treatment due to its antimicrobial and antibiofilm capabilities [324]. However, because NO has a short half-life and limited diffusion, its efficacy is highly dependent on local concentration and exposure duration at lesion sites. Controlled loading and stimuli-responsive release in pathological microenvironments remain key technical challenges [325]. To address this, Zheng et al. [215] constructed an engineered PBzyme library containing eight different metal dopants and screened ruthenium-doped PBzyme for its superior antioxidant activity and photothermal performance (Fig. 9d). Sodium nitroprusside was then incorporated into the optimized PBzyme via in situ loading at room temperature to obtain SPBzyme. The released nitrogen oxides enhanced antibacterial efficacy through NO gas therapy, overcoming biofilm-associated drug resistance. The study demonstrated that SPBzyme effectively eradicated bacterial biofilms via photothermal ablation and exhibited excellent radical scavenging and anti-inflammatory activity at the cellular level. This work not only validated the antibacterial and anti-inflammatory potential of SPBzyme but also provided a methodological reference for the construction and screening of metal-engineered nanozyme libraries.

From the perspective of restoring cellular homeostasis in periodontal therapy, Zhu et al. [321] developed a mesoporous MOF-based nanozyme, MnO_2_@UiO-66(Ce). This nanozyme not only rapidly scavenges ROS through cascade SOD/CAT-like activities, but also activates the Mn^2+^-mediated SIRT1-FOXO3-BNIP3 pathway, enhancing mitochondrial autophagy and promoting the removal of damaged mitochondria, thereby re-establishing long-term cellular homeostasis. In vitro studies demonstrated its capability to restore mitochondrial balance and promote osteogenic differentiation of periodontal ligament cells (Fig. 9e).

In terms of responsive drug release and tissue regeneration, Li et al. [322] designed a yolk-shell structured copper-ruthenium-based nanozyme (Cu_2_O@RuO_2_, CRNC), integrating antioxidant, osteogenic, and angiogenic functionalities. The RuO_2_ outer shell provides robust ROS-scavenging ability and promotes M2 macrophage polarization, thereby attenuating inflammation. Meanwhile, the Cu_2_O core serves as a responsive Cu^2+^ reservoir that releases ions under ROS- and pH-rich conditions at the lesion site. The released Cu^2+^ ions activate TGF-β/PI3K and HIF-1α signaling pathways, respectively enhancing angiogenesis and osteogenic repair (Fig. 9f). Qu et al. [323] further developed an injectable NIR-driven thermoresponsive hydrogel system (AgZ@Au/PLEL) for periodontal regeneration (Fig. 9g). In this system, Au nanoparticles with GOx-like activity catalyze glucose in periodontal pockets to produce H_2_O_2_ and generate an acidic environment, both of which enhance catalytic efficiency and photothermal bactericidal effects under NIR stimulation. The acidic microenvironment triggers the degradation of ZIF-8, thereby releasing Zn^2+^ ions, which promote osteogenic differentiation. The hydrogel exhibits excellent injectability and shape adaptability, making it suitable for irregular periodontal defects. In vitro experiments revealed that this platform achieved efficient photothermal-assisted antibacterial performance and induced osteogenesis in bone marrow-derived mesenchymal stem cells, demonstrating great potential for precise antibacterial therapy and tissue regeneration.

### 6.5 Joint inflammation

#### 6.5.1 Osteoarthritis

OA is a degenerative disease primarily caused by articular cartilage damage and is often accompanied by subchondral sclerosis, synovial inflammation, and periarticular soft tissue lesions [85,326]. Due to its high prevalence and disability rate, OA has become a major public health concern [327]. Studies have shown that oxidative stress plays a pivotal role in OA pathogenesis, while orally administered small-molecule antioxidants have limited therapeutic effects due to their poor ability to penetrate the joint barrier and typically require frequent intra-articular injections [328]. In contrast, nanozymes—with their superior stability, tunability, and targeting capabilities—can effectively traverse the joint barrier and exert sustained antioxidant activity, showing great promise in OA treatment.

**Fig. 10:**
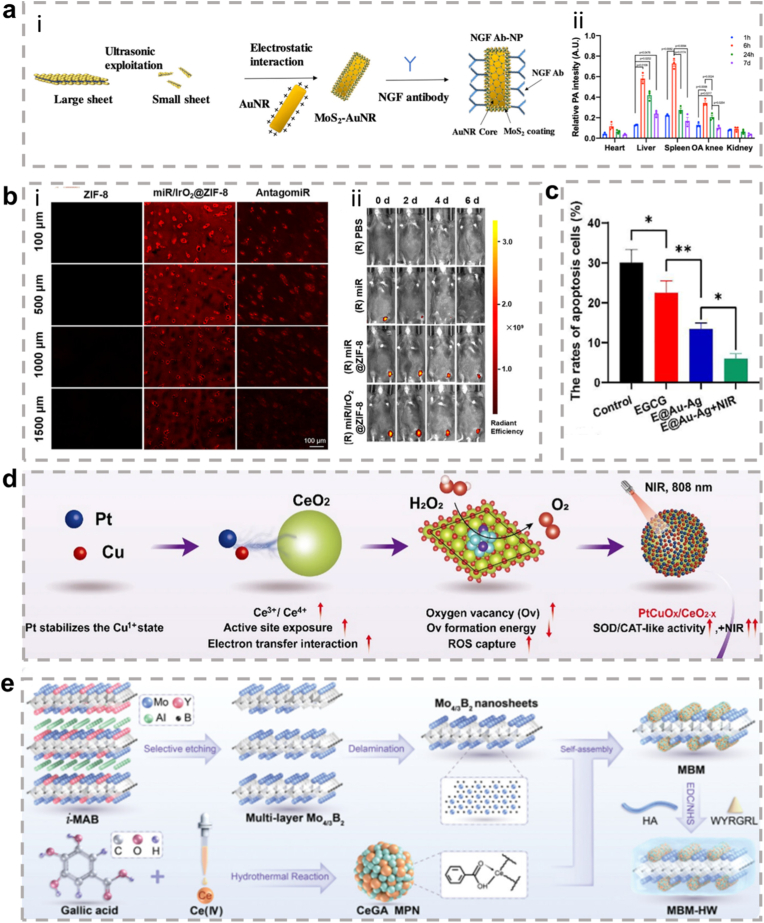
(a) Gold nanorods coated with molybdenum disulfide nanosheets for osteoarthritis pain treatment: (i) Schematic illustration of MoS_2_-AuNR synthesis; (ii) Semi-quantitative biodistribution of MoS_2_-AuNR in major organs of mice as determined by corresponding PA signal intensity [329]. Copyright 2021 American Chemical Society. (b) miR/IrO_2_@ZIF-8 platform for synergistic osteoarthritis therapy: (i) Two-photon excitation images of human articular cartilage tissue incubated with miR/IrO_2_@ZIF-8 (100 μg/mL) and Cy5-antagomiR-181a (200 nM) at different depths (scale bar: 100 μm); (ii) Representative time-dependent biodistribution images at 0, 2, 4, and 6 days after intra-articular injection of ZIF-8, Cy5-antagomiR-181a, miR@ZIF-8, and miR/IrO@ZIF-8 [17] Copyright 2024 The Author(s). (c) Quantitative analysis of chondrocytes after different treatments by flow cytometr [330]. Copyright 2022 Acta Materialia Inc. (d) Schematic illustration of PtCuOX/CeO_2__-__x_ synthesis [331]. Copyright 2025 BioMed Central Ltd. (e) Schematic diagram of MBM-HW synthesis [332]. Copyright 2025 Wiley-VCH GmbH. (For interpretation of the references to color in this figure legend, the reader is referred to the Web version of this article.)

Ou et al. [329] developed a nerve growth factor (NGF)-targeted gold nanorod-based nanozyme (MoS_2_-AuNR) derived from two-dimensional MoS_2_. Photoacoustic imaging (PA) demonstrated that the NGF monoclonal antibody-conjugated nanoprobe (anti-NGF-MoS_2_-AuNR) actively targeted OA-affected knee joints. ASOs targeting miR-181a-5p can downregulate genes involved in cartilage catabolism and chondrocyte apoptosis, thereby alleviating cartilage destruction (Fig. 10a). However, challenges such as poor stability, off-target effects, and low intracellular uptake hinder the therapeutic efficacy of ASOs. To overcome these obstacles, Wu et al. [17] pioneered a biocompatible MOF-encapsulated nanozyme (miR/IrO_2_@ZIF-8) as an advanced delivery platform for ASOs, which presented a paradigm shift from conventional delivery systems. The key innovation lies in the unique core-shell structure where the IrO_2_ nanozyme core provides multifaceted catalytic activity, while the ZIF-8 MOF shell acts as a exceptionally protective matrix and a stimuli-responsive gatekeeper. This design fundamentally overcomes the critical bottlenecks of traditional ASO therapy: the IrO_2_@ZIF-8 system not only effectively shields the encapsulated antagomiR-181a from nuclease degradation, drastically improving its in vivo stability and half-life, but also enables a controlled, pH-responsive release within the acidic pathological environment of OA joints, thereby minimizing off-target effects. A groundbreaking advantage was visualized via two-photon imaging, which confirmed the nanozyme's unprecedented capacity for deep cartilage penetration, reaching depths of up to 1.5 mm in human cartilage (Fig. 10b). Furthermore, the nanozyme's surface chemistry and size facilitated efficient cellular uptake and subsequent lysosomal escape, ensuring the therapeutic cargo reached its cytoplasmic target. Coupled with its long-term retention in OA joints—with 18.26 % of the initial signal persistently detectable six days post-injection, significantly extending the therapeutic window and reducing dosing frequency—this nanozyme platform demonstrated a synergistic combination of outstanding multienzyme catalytic activity, deep tissue penetration, and intelligent drug release performance. This comprehensive set of capabilities positions it as a far superior and more effective strategy for the treatment of OA compared to the direct application of free ASOs. Xu et al. [330] introduced a multifunctional NIR-sensitive heterostructure—epigallocatechin gallate (EGCG)-decorated Au-Ag nanocages (E@Au-Ag)—as an enzyme-responsive nano-platform for OA therapy. Under NIR irradiation, intra-articular injection of E@Au-Ag raised joint cavity temperatures to 46.6 °C, facilitating the release of EGCG to promote cartilage regeneration. Molecular biology results indicated that E@Au-Ag possessed inherent antioxidant activity, reducing chondrocyte apoptosis by up to 83.3 % (Fig. 10c).

The limited enzymatic activity of CeO_2_ nanozymes has restricted their effectiveness in treating OA. Enhancing their catalytic performance by increasing oxygen vacancies has shown promise. Yang et al. [331] introduced bimetallic copper and platinum into CeO_2_ to synthesize PtCuO_x_/CeO_2__-__x_, where Cu and Pt significantly enhanced the oxygen vacancy concentration and increased the Ce^3+^/Ce^4+^ ratio. Meanwhile, CeO_2_ facilitated the uniform dispersion of Cu and Pt nanoparticles (Fig. 10d). This nanozyme promoted electron transfer by lowering the formation energy of oxygen vacancies, increased intermediate adsorption energy, and reduced activation energy, thereby exhibiting high SOD- and CAT-like activity. Additionally, it demonstrated excellent photothermal conversion efficiency, contributing to its potent therapeutic effect in OA. Similarly, Yu et al. [333] developed a dual-biomimetic photothermal nanozyme—MoS_2_@PDA-Mg@PSB (MPMP)—comprising Mg^2+^- MoS_2_ doped with polydopamine and coated with polysulfobetaine. This nanozyme also proved effective in photothermal therapy for OA.

In addition to their mimetic enzymatic activities, some nanozymes—when rationally engineered into multifunctional heterojunctions—can alleviate oxidative stress in chondrocytes, preserve mitochondrial function, inhibit cartilage matrix degradation and ferroptosis, and ultimately slow OA progression. Zhang et al. [332] fabricated a bio-heterojunction nanozyme (MBM-HW) by self-assembling 2D Mo_4_/_3_B_2__-_x MBene, a cerium-gallic acid metal-polyphenol network (MPN), and a cartilage-targeting shell composed of hyaluronic acid and WYRGRL peptide (HW) (Fig. 10e). This nanozyme exhibited SOD-, CAT-, and GPx-like activity, effectively scavenging excessive ROS. It also demonstrated dual-responsive drug release and cartilage-targeting capabilities. Mechanistically, MBM-HW was shown to attenuate the Perk/eIF2α signaling cascade associated with endoplasmic reticulum stress, suppress ECM degradation and ferroptosis, and maintain chondrocyte homeostasis.

#### 6.5.2 Rheumatoid arthritis

Rheumatoid arthritis (RA) is an autoimmune disease that can lead to progressive joint dysfunction in severe cases [334]. RA can be treated via two opposing catalytic pathways: one involves antioxidant activities (e.g., SOD-like, CAT-like, and GPx-like) to reverse the pro-inflammatory phenotype of macrophages; the other utilizes pro-oxidant activities (e.g., POD-like and OXD-like) to directly eliminate inflammatory effector cells.

**Fig. 11:**
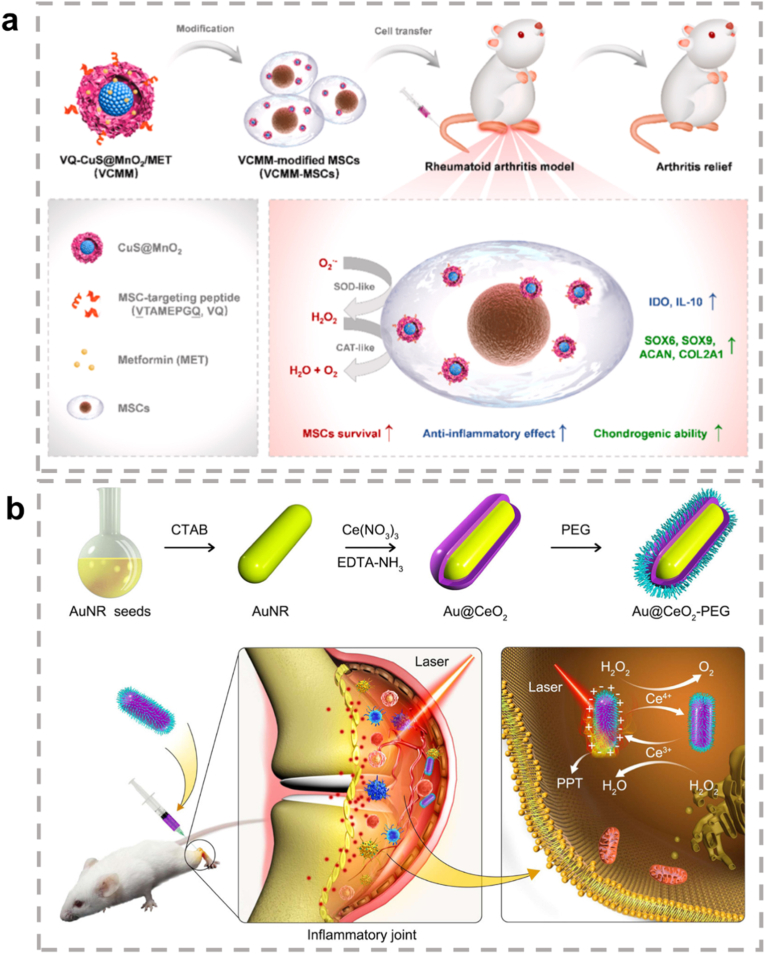
(a) Schematic illustration of the preparation of MSC-modified VQ-CuS@MnO_2_/MET nanoparticles and their application in the treatment of RA [335]. Copyright 2022 Elsevier Ltd. All rights reserved. (b) Schematic diagram of the synthesis and therapeutic application of Au@CeO_2_ nanoparticles [336]. Copyright 2020 American Chemical Society.

Cu and Mn are involved in the formation of natural superoxide dismutase and promote chondrogenesis of stem cells, thus treating RA through antioxidant activity. Based on this, Lu et al. [335] synthesized CuS@MnO_2_ nanoparticles, modified with a MSC-targeting peptide (VTAMEPGQ, referred to as VQ) and loaded with metformin (MET), which enhances mesenchymal stem cell (MSC) anti-inflammatory activity, resulting in the preparation of VQ-CuS@MnO_2_/MET NPs (abbreviated as VCMM-MCS). VCMM-MCS exhibited anti-inflammatory effects, promoted chondrogenesis, and improved cell survival under oxidative stress (Fig. 11a). Intravenous administration reduced synovial hyperplasia and cartilage damage, effectively relieving arthritis syndromes in CIA and AIA models. Treating RA through pro-oxidative activity is also a promising strategy. Wang et al. [336] prepared a PEG-modified ceria-coated gold nanorod (Au@CeO_2_), which can combine local photothermal and oxygen-enriched therapy to eliminate inflammatory cells in affected joints (Fig. 11b). Upon laser irradiation, Au@CeO_2_ exhibits exponential photothermal enhancement through localized surface plasmon resonance. The generated heat not only eradicates hyperproliferative inflammatory cells but also facilitates the Ce^4+^ to Ce^3+^ transition, significantly enhancing cerium's catalase-like activity and accelerating H_2_O_2_ decomposition to release oxygen, thereby alleviating hypoxia. Furthermore, Au@CeO_2_ markedly improved RA lesions and effectively suppressed the expression of pro-inflammatory cytokines and hypoxia-inducible factors under combined thermal and oxygen therapy.

### 6.6 Inflammatory skin diseases

Inflammatory skin diseases, especially chronic wounds associated with diabetes, often lead to persistent tissue damage, chronic inflammation, and functional impairment. These complications severely affect patients’ quality of life and impose a heavy burden on healthcare systems [337]. Diabetic chronic wounds, as a typical example of non-healing injuries, are particularly difficult to treat due to multiple pathological factors. These include oxidative stress caused by the accumulation of ROS, local tissue hypoxia, metabolic disorders triggered by hyperglycemia, and bacterial biofilm infections, all of which significantly impair natural healing capacity. Consequently, the development of intelligent therapeutic platforms capable of coordinated regulation of multiple factors has become a key research focus.

**Fig. 12:**
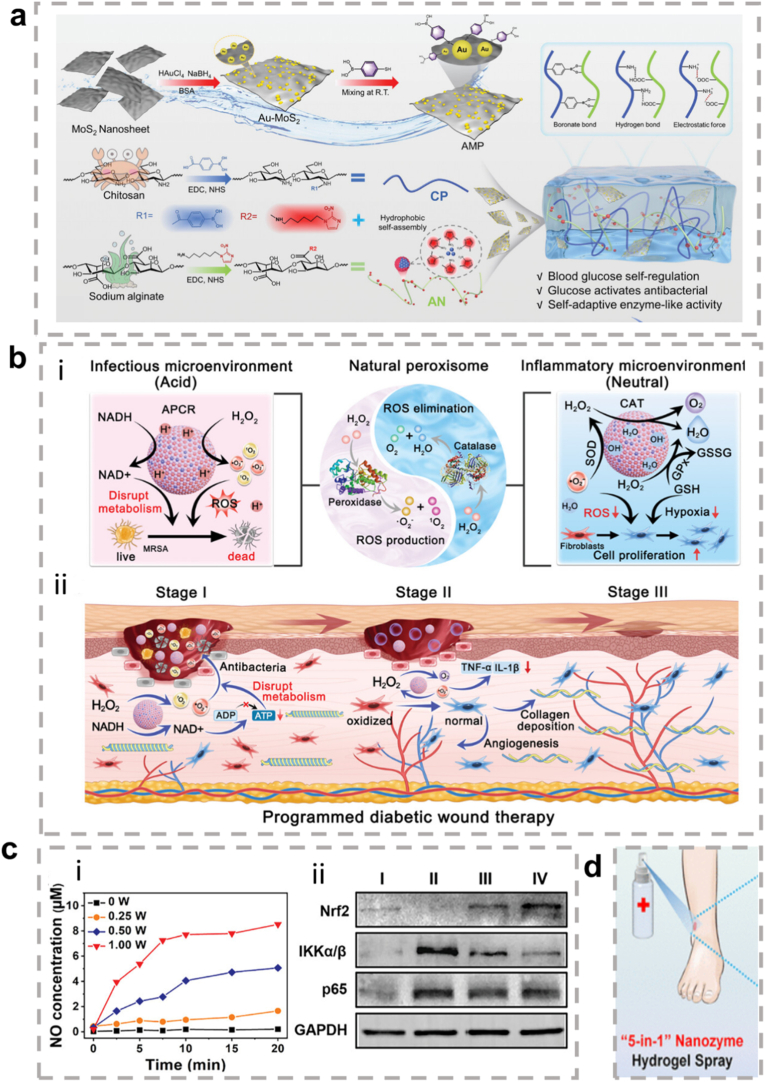
(a) Schematic illustration of the synthesis of a glucose-activated programmable hydrogel for infected diabetic wound healing, comprising AMP, CP, and AN components [338]. Copyright 2025 Wiley-VCH GmbH.(b) Design and application of self-adaptive artificial peroxisomes for diabetic ulcer therapy. (i) Bioinspired design strategy of the wound microenvironment-responsive artificial peroxisomes with synergistic Co-Ru dual active centers and pH-switchable enzyme-like activities. (ii) Illustration of the therapeutic mechanism of APCR for programmed diabetic wound therapy [342]. Copyright 2024 Wiley-VCH GmbH. (c) UAPsBP@Gel for the treatment of infected diabetic ulcers. (i) NO release curves of UAPsBP under different power densities. (ii) Western blot analysis of Nrf2, IKKα/β, and p65 proteins related to the Nrf2/NF-κB signaling pathway [343]. Copyright 2025 Wiley-VCH GmbH. (d) Multifunctional ACPCAH spray for DFU wound healing against multidrug-resistant bacterial infections [344]. Copyright 2023 American Chemical Society.

Hydrogels have been widely employed as carrier materials for functional nanozymes in the treatment of diabetic wounds due to their excellent biocompatibility, moisture retention, and tunable composition. Yang et al. [338] developed a programmable hydrogel for the intelligent regulation and treatment of diabetic wounds. This hydrogel comprises multi-enzyme-like Au-MoS_2_-phenylboronic acid nanozymes (AMP), insulin-loaded nitroimidazole-modified alginate microcapsules (AN), and pentylboronic acid-modified chitosan (CP), integrated into a complete system through multiple interactions (CPAN-AMP) (Fig. 12a). Under hyperglycemic conditions, AMP catalyzes glucose oxidation to generate ROS for antibacterial activity, while hypoxia simultaneously induces insulin release from AN microcapsules, enabling blood glucose regulation for up to 12 h. Under normoglycemic conditions, AMP switches to oxygen-producing activity, suppressing insulin release and alleviating tissue hypoxia. This hydrogel achieves synergistic antibacterial, glycemic, and hypoxia modulation through dynamic blood glucose-responsive mechanisms, resulting in a wound healing rate approximately three times that of conventional dressings. Due to their excellent physicochemical properties and compatibility with hydrogels, Au-Pt bimetallic nanozymes have become a research hotspot in diabetic wound treatment. Zhang et al. [339]integrated Au-Pt nanoparticles into a self-healing hydrogel (OHCN), significantly promoting wound repair. Qi et al. [340] constructed an AuPt@melanin-GHM3 hydrogel combined with a mild photothermal therapy strategy to accelerate glucose consumption and ROS clearance, while promoting collagen deposition and angiogenesis. Hui et al. [162] used microfluidic technology to develop multifunctional composite microspheres (GMAP) combining Au@Pt NPs and GelMA hydrogel. In hyperglycemic environments, these microspheres protect bone marrow mesenchymal stem cell (BMSC) function and enhance bone regeneration, offering a novel solution for diabetes-related bone defects. In addition to therapeutic applications, Au-Pt nanozymes can also be employed for glucose monitoring. Kim et al. [341] developed HA-Au@Pt BiNCs-based smart contact lenses capable of sensitively responding to blood glucose changes for non-invasive monitoring.

Long-term diabetes can lead to the development of diabetic foot ulcers (DFUs), which are often accompanied by bacterial infection, persistent inflammation, and impaired angiogenesis [345]. Traditional antibiotics are often ineffective against drug-resistant bacteria, making the development of nanozyme systems with coordinated antibacterial, antioxidative, and pro-angiogenic functions an important research direction.

Gao et al. [342] synthesized an artificial peroxisome (APCR) with Co-Ru dual active sites via a hydrothermal method. Its catalytic mechanism is based on intermediate adsorption/desorption behavior, where the interaction between metal sites and reactants is critical to its high activity. APCR can switch between POD- and CAT-like activities under different pH conditions, enabling excellent ROS regulation (Fig. 12b). DFT analysis revealed that Co sites promote H_2_O_2_ adsorption, while Ru sites facilitate intermediate desorption, thus enhancing the efficiency of diatomic catalysis.

Guo et al. [346] found that PtCuTe trimetallic nanozymes exhibited stronger ROS scavenging and antibacterial activity than traditional PtCu bimetallic systems. These nanozymes also promoted intercellular crosstalk among different cell types, forming a positive feedback loop to facilitate angiogenesis. Furthermore, PtCuTe stimulated macrophage polarization toward the M2 phenotype and improved fibroblast migration.

In terms of combinatorial therapies, Ma et al. [347] constructed a MoS_2_-CeO_2_ nanocomposite that combines the photothermal antibacterial activity of MoS_2_ with the ROS-scavenging ability of CeO_2_, demonstrating notable anti-inflammatory and antibacterial effects. Zhao et al. [348] fabricated Au nanocluster-modified zirconium-based porphyrinic MOFs (Au NCs@PCN) via an in situ growth method. Au NCs@PCN function as ROS generators rather than scavengers, contributing to bacterial eradication. These nanomaterials also displayed excellent photothermal effects, destroying bacterial membranes and inducing substantial protein leakage under NIR irradiation. Immunoblotting analysis confirmed their ability to promote angiogenesis and upregulate epithelial cytokines such as VEGF and EGF, demonstrating their therapeutic potential in diabetic wound healing.

Meng et al. [343] developed an injectable hybrid hydrogel system (UAPsBP@Gel), composed of a Ui MOFs, an AuPd nanoshell, the photosensitizer BNN6, and PEG@Gel. As a NO reactor,the system triggers NO release via the photothermal effect of the AuPd nanoshell under NIR-II irradiation, enabling synergistic elimination of biofilms in deep infectious DFUs (Fig. 12c). The AuPd nanozyme possesses SOD, GOx, and CAT-like activities, allowing for cascade ROS and glucose scavenging. It also remodels the wound microenvironment, activates the Nrf2/HO-1 pathway, inhibits NF-κB signaling, and induces macrophage polarization toward the anti-inflammatory M2 phenotype. The hydrogel further enhances hemostasis, cell migration, angiogenesis, and NO delivery, significantly accelerating wound healing and showcasing great potential for multi-stage modulation in treating infectious DFUs.

In addition to PDT,CDT has emerged as a promising strategy that induces local Fenton reactions to eliminate pathological cells, thereby enhancing therapeutic outcomes [349]. Xu et al. [344] developed Ti-MnO_2_-CPA@Ce6 cascade nanozymes, which serve as ultrasound-responsive, degradable hydrogels for CDT-based treatment of diabetes-related implant infections. Building on this, Shang et al. fabricated a multi-component phosphorus-doped graphitic carbon nitride nanosheet (ACPCAH) loaded with L-arginine, gold nanoparticles, and Cu_1_._6_O. This nanozyme system exhibits five enzyme-mimicking activities (SOD, CAT, GOx, POD, and NOS), broad-spectrum ROS scavenging capability, and pronounced ultrasound responsiveness (Fig. 12d).

BAIs are a leading cause of implant failure and are often accompanied by persistent immune activation and chronic inflammation. When occurring in diabetic or orthopedic settings, BAIs can result in devastating consequences [350]. Biofilm formation provides a physical barrier that enhances bacterial resistance and continuously stimulates the host immune system, triggering localized inflammation and exacerbating tissue damage. Although traditional antibiotics can temporarily suppress bacterial growth, their inability to penetrate biofilms and the risk of inducing resistance limit clinical efficacy. Nanozymes, as a new generation of antimicrobial agents, exhibit broad-spectrum antibacterial activity and negligible resistance development, showing great potential for combined antibacterial and anti-inflammatory treatment of BAIs [351].

**Fig. 13:**
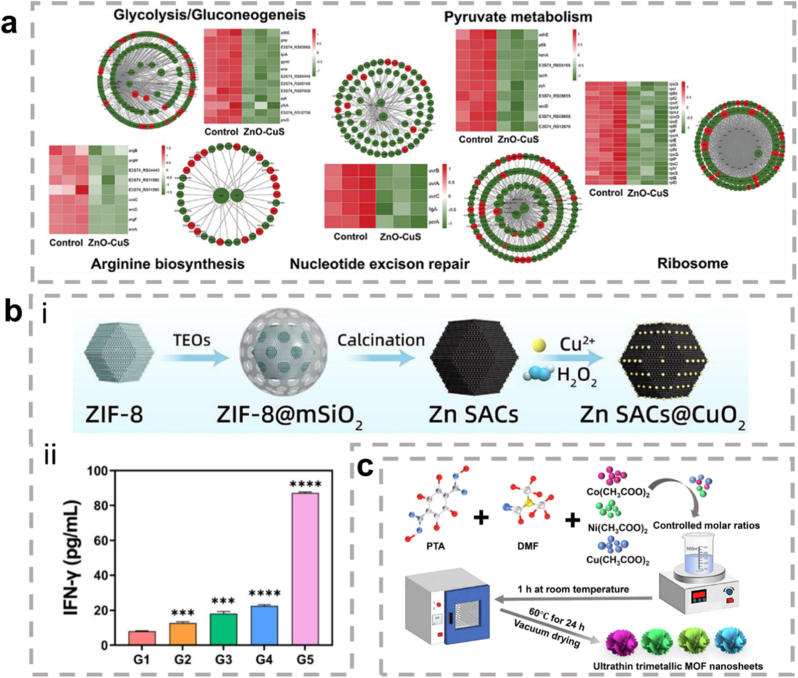
(a) Protein-protein interaction (PPI) network diagram and corresponding heatmap illustrating the antibacterial mechanism of ZnO-CuS [352]. Copyright2024 Wiley-VCH GmbH. (b) Zn SACs@CuO_2_ nanozyme for wound healing in biofilm infections. (i) Schematic illustration of the synthesis of Zn SACs@CuO_2_ NPs. (ii) Expression levels of IFN-γ in wound tissues after different treatments, as measured by ELISA. The five treatment groups are: PBS (G1), H_2_O_2_ + NIR (G2), Zn NPs + H_2_O_2_ + NIR (G3), Zn SACs + H_2_O_2_ + NIR (G4), Zn SACs@CuO_2_ + H_2_O_2_ + NIR (G5); NIR conditions: 808 nm, 1.0 W cm^−2^, 5 min [181]. Copyright 2023 Elsevier B.V. All rights reserved. (c) Schematic of the synthesis of (Ni_2_Co_1_)_1__−__x_Cu_x_ via a one-pot ambient-temperature solution-phase method [353]. Copyright 2022 Elsevier Inc. All rights reserved.

To improve antibacterial efficacy and targeting, Zhang et al. [352] developed a ZnO-CuS nanoflower-like structure. ZnO possesses POD and GPx-like activity, disrupts metal ion homeostasis, and interferes with the metabolism of amino acids such as phenylalanine, tyrosine, and tryptophan, enabling direct bactericidal effects. However, its surface lacks charge, limiting its adhesion to bacterial membranes. To enhance targeting ability, positively charged CuS was introduced to facilitate electrostatic interactions with the peptidoglycan amino groups of bacterial cell walls. Moreover, Cu^2+^ significantly inhibits the metabolism of key amino acids including glycine, arginine, and serine, thus enhancing both antibacterial and anti-inflammatory outcomes (Fig. 13a). Considering that implant-associated infections are often accompanied by tissue necrosis and cavity formation, simple injection of nanozyme solutions is insufficient to maintain therapeutic concentrations. Sun et al. [181] further encapsulated the ZnO-CuS complex in a thermosensitive F127 hydrogel to construct ZnO-CuS/F127 nanozymes. This system not only suppresses MRSA metabolism (including arginine synthesis, nucleotide excision repair, energy metabolism, and protein biosynthesis) but also promotes M2 macrophage polarization and tissue regeneration (Fig. 13b).

Zhou et al. [354] designed a core-shell nanozyme CeO_2_@ZIF-8/Au, capable of both generating and scavenging ROS in a self-regulated manner to enable integrated bactericidal, anti-inflammatory, and wound-healing effects. The Au outer layer of CeO_2_@ZIF-8/Au exhibits POD-like activity that facilitates ROS accumulation for bacterial killing. Upon degradation of the ZIF-8 MOF shell in acidic microenvironments, encapsulated CeO_2_ is gradually released, exerting SOD and CAT activities to scavenge excess ROS. This autonomous ROS-regulating nanozyme offers a promising strategy for precision treatment of BAIs.From the perspective of reusability, scalable synthesis, and functional integration, efforts have been made to enhance the practicality of antibacterial nanozymes. Ding et al. [355] developed Fe_3_O_4_/CuO_x_ nanozymes with robust POD-like activity, retaining high catalytic performance within 25–60 °C and effectively eliminating *Staphylococcus aureus* and *Escherichia coli* under low H_2_O_2_ concentrations. Additionally, these nanozymes can be magnetically separated and reused at least five times, demonstrating excellent stability and cost-effectiveness. To further improve production efficiency, Lin et al. [353] synthesized ultrathin layered trimetallic MOF nanosheets ((Ni_2_Co_1_)_0_._5_Cu_0_._5_) under ambient conditions (Fig. 13c). The superlattice structure of the NiCo-MOF shortens electron transfer and ion diffusion pathways, increases surface area, and provides abundant redox-active sites. Copper ions serve as cofactors to stabilize the nanozyme conformation, neutralize anions in the microenvironment, reduce electrostatic repulsion, and accelerate electron transfer to enhance POD-like activity. The flexible adjustment of metal ratios also offers scalability and optimization potential for large-scale applications.

## 7 Conclusions and future directions

This review systematically summarizes the latest advances in metallic hybrid nanozymes for advanced inflammatory disease therapy. Throughout the text, several specific metallic hybrid systems and design strategies have demonstrated significant clinical translation potential due to their exceptional performance. Among them, systems capable of mimicking superoxide dismutase/catalase cascade reactions (such as Pt@PCN222-Mn [22] and various manganese-based Prussian blue analogs [215]) show remarkable effectiveness in scavenging reactive oxygen species. Single-atom and dual-atom nanozymes with well-defined and high-density active sites (e.g., FeMn-DAN [165]) maximize catalytic efficiency. Furthermore, multifunctional heterostructures that integrate nanozymes with physical therapies such as photothermal and sonodynamic therapies (e.g., TPP-Au-Ru [101], MoS_2_-AuNR [329]) provide powerful platforms for synergistic treatment. At the rational design level, strategies including multi-metal synergy, valence state engineering, defect engineering, and intelligent structural control (e.g., core-shell and Janus structures) enable precise regulation of the electronic structure and microenvironment responsiveness of nanozymes, thereby achieving targeted intervention in specific inflammatory signaling pathways. These systems and strategies represent the current frontier in metallic hybrid nanozyme research and form a solid foundation for future clinical applications. Despite the remarkable anti-inflammatory efficacy demonstrated in various disease models, metallic hybrid nanozymes still face critical challenges. We believe that overcoming these hurdles will catalyze a transformative shift and inject renewed momentum into the field of nanozyme research.

(1) Precise modulation of catalytic activity

The active sites of nanozymes have been widely studied to guide the design of modulated structures for improved activity [19]. At present, the field remains concentrated on mimicking POD, CAT, SOD, and OXD activities; broadening this spectrum is critical for fulfilling varied therapeutic needs. Ultimately, engineering multifunctional composite materials around metallic hybrid nanozymes—even with greater synthetic complexity—will markedly elevate their functional capacity and biomedical applicability, thereby paving the way for sophisticated multi-target combination therapies.

(2) Transitioning to a data-driven design paradigm

The research paradigm for nanozymes is shifting from traditional experience-driven approaches to a data-driven design framework. This transition relies on the integration of chemistry and artificial intelligence. Unlike conventional enzyme kinetic parameters, micro-level descriptors derived from catalytic chemistry—such as adsorption energy, d-band center, and reaction Gibbs free energy—provide fundamental insights into catalytic activity from electronic and geometric structure perspectives, offering critical guidance for rational design. Building on this foundation, the combination of high-throughput calculations and machine learning promises to establish large-scale structure-activity databases, enabling accelerated screening of material combinations and active site configurations, as well as accurate prediction of high-performance structures. Integrating automated closed-loop synthesis systems with AI-assisted optimization and high-throughput data analytics will pave the way for scalable production of metallic hybrid nanozymes with predictable properties, ushering in a new era of efficient and reliable analytical chemistry.

Notably, while current research provides comprehensive analysis of POD-like nanozymes, the mechanistic exploration of SOD- and CAT-type enzymes remains insufficient, particularly lacking unified descriptor systems to correlate their metal center structures with catalytic properties. Furthermore, although theoretical calculations demonstrate considerable potential in the field of metallic hybrid nanozymes, successful translation of predictive results into practical materials via automated synthesis platforms remains limited. Future efforts should prioritize establishing dedicated descriptor systems for SOD and CAT activities, and strengthening the connection between theoretical design and experimental validation to achieve a complete transition from computational prediction to practical application.

(3) In-depth exploration of catalytic mechanisms

Although metallic hybrid nanozymes demonstrate versatile potential, studies elucidating their specific catalytic mechanisms of ROS remain limited and often focus on individual pathways or molecules. There is a lack of comprehensive and systematic understanding of the causal relationship between ROS and inflammation, as well as the intricate regulatory networks involved. Future research should focus on ROS signaling networks to reveal their multifaceted roles in inflammation under different pathological contexts, thereby guiding the rational design of more efficient nanozymes.

(4) Integrated multi-omics analysis

While current research on metallic hybrid nanozymes has largely centered on ROS regulation and intracellular signaling pathways, a critical challenge remains in correlating their well-documented in vitro enzyme-like activity with actual therapeutic outcomes in vivo. This gap underscores the need to not only refine our understanding of their localized actions but also to investigate their broader biological interactions. Crucially, bridging this gap requires a deliberate selection of nanozymes with specific catalytic profiles tailored to the pathophysiology of the target disease. For instance, nanozymes exhibiting high SOD-like and CAT-like activities are ideally suited for managing sterile inflammatory diseases. These enzymes work in concert to dismantle the ROS cascade—SOD converts ·O_2_^−^ into H_2_O_2_, which CAT then decomposes into harmless H_2_O and O_2_—thereby alleviating oxidative stress and suppressing subsequent NF-κB and NLRP3 inflammasome activation. Conversely, in scenarios involving bacterial infection-related inflammation, nanozymes with prominent POD-like and OXD-like activities are more advantageous. They leverage the typically elevated H_2_O_2_ levels at infection sites to catalyze the generation of highly toxic ·OH or other reactive species, which not only directly eradicate pathogens but also concurrently disrupt the inflammatory microenvironment fostered by the infection. Therefore, the future rational design of metallic hybrid nanozymes must transcend the mere pursuit of high activity in vitro. It should strategically align the enzyme-mimicking profile with the specific redox landscape of the target disease, while also meticulously considering in vivo delivery, biodistribution, and potential long-term biocompatibility to truly translate catalytic potency into definitive therapeutic efficacy.

Beyond these mechanistic and translational challenges, the systemic biological effects of nanozymes—such as their potential influence on the gut microbiome or endocrine metabolic pathways—are still insufficiently explored. A broader investigative scope is essential to fully elucidate their in vivo behavior, long-term biological impacts, and overall safety profile.

Of particular concern in this regard is long-term biosafety. Future studies should employ integrated multi-omics approaches—spanning transcriptomics, metabolomics, proteomics, and microbiomics—to systematically evaluate whether nanozyme interventions induce unintended immunosuppressive effects resembling glucocorticoid action or disrupt the homeostasis of key inflammatory pathways such as NF-κB and JAK-STAT.

By leveraging multi-omics data to decode molecular networks and causal mechanisms within the inflammatory microenvironment, researchers can bridge the gap between laboratory evidence and clinical efficacy. This systems-level understanding will not only clarify nanozyme mechanisms and safety but also help tailor more precise and personalized anti-inflammatory strategies.

(5) Ensuring biosafety and facilitating clinical translation

As metallic hybrid nanozymes find expanding applications in biomedicine, their biosafety remains a pivotal challenge for clinical translation. The main toxicity concerns originate from their slow metabolic clearance and inadequate biodegradability, resulting in progressive accumulation in organs such as the liver, spleen, and kidneys. Such accumulation may provoke chronic toxicity, elicit immune reactions, and disrupt normal cellular activities. Furthermore, physiological factors—including pH variations, biomolecular adsorption, redox conditions, and immune clearance—can lead to nanozyme deactivation, off-target effects, and metal ion leakage, which collectively hinder clinical adoption.

Enhancing targeting specificity is equally crucial to minimize off-target toxicity and lower therapeutic dosages. Strategies such as receptor-specific modification, stimulus-responsive designs, and engineered cellular delivery can improve site-specific accumulation while reducing systemic exposure. Additionally, overcoming physiological barriers—including the blood-brain barrier and synovial membrane—by developing nanozymes with superior tissue penetration capability represents an urgent research direction for expanding their therapeutic utility.

To overcome these limitations, future efforts should focus on designing metallic hybrid nanozymes with improved biodegradability and renal-clearable dimensions to promote efficient elimination. Furthermore, it is essential to establish a comprehensive safety evaluation framework that systematically assesses pharmacokinetics, biodistribution, immunogenicity, and long-term biological fate. Such a system would offer a scientific foundation for evaluating nanozyme safety across tissues and applications.

## CRediT authorship contribution statement

**Xueqian Xia:** Writing – original draft, Validation, Software, Methodology, Investigation, Formal analysis, Data curation, Conceptualization. **Xiang Liu:** Writing – original draft, Validation, Software, Resources, Methodology, Data curation. **Yue Gao:** Writing – original draft, Software, Methodology, Investigation, Data curation. **Jiatong Lin:** Validation, Methodology, Investigation. **Shuangxue Pan:** Methodology, Investigation, Data curation. **Weijian Cheng:** Writing – review & editing, Methodology. **Sheng Huang:** Visualization, Resources, Methodology, Investigation. **Xingyue Liu:** Writing – review & editing, Visualization, Validation, Supervision, Investigation, Conceptualization. **Jia-Wei Shen:** Writing – review & editing, Supervision, Resources, Project administration, Investigation, Funding acquisition, Formal analysis. **Wei Duan:** Writing – review & editing, Visualization, Validation, Supervision, Software, Resources, Project administration, Methodology, Investigation, Funding acquisition, Formal analysis, Data curation, Conceptualization.

## Declaration of competing interest

The authors declare that they have no known competing financial interests or personal relationships that could have appeared to influence the work reported in this paper.

## Data Availability

Data will be made available on request.
